# Supplementary material for: C-terminal modification and functionalization of proteins via a self-cleavage tag triggered by a small molecule
Source: Nat Commun. 2023 Nov 7;14:7169. doi: 10.1038/s41467-023-42977-x (PMC10630284; doi:10.1038/s41467-023-42977-x)

Supplementary Information for:

C-terminal Modification and Functionalization of Proteins via A Self-cleavage Tag Triggered by A Small Molecule

Yue Zeng[1,2,5], Wei Shi[1,5], Zhi Liu[1,3], Hao Xu[3], Liya Liu[1], Jiaying Hang[1], Yongqin Wang[4], Mengru Lu[4], Wei Zhou[4], Wei Huang[1,2,3,4], Feng Tang[1,2,4]*

[1]State Key Laboratory of Drug Research, Center for Biotherapeutics Discovery Research, Shanghai Institute of Materia Medica, Chinese Academy of Sciences, No.555 Zuchongzhi Rd, Pudong, Shanghai 201203, China.

[2]University of Chinese Academy of Sciences, No.19A Yuquan Road, Beijing 100049, China.

[3]School of Chinese Materia Medica, Nanjing University of Chinese Medicine, No. 138 Xianlin Rd, Nanjing 210023, China.

[4]School of Pharmaceutical Science and Technology, Hangzhou Institute of Advanced Study, Hangzhou 310024, China.

[5]These authors contributed equally: Yue Zeng, Wei Shi.

*Corresponding author: tangfeng2013@simm.ac.cn (Feng Tang)

Contents

1. Supplementary figures and tables……………………………………………………...2
2. General Information and procedures……………………………………………………31
3. Experimental section…………………………………………………………………...35
4. Protein sequences……………………………………………………………………..40
5. NMRs…………………………………………………………………………………..47

1. Supplementary figures and tables

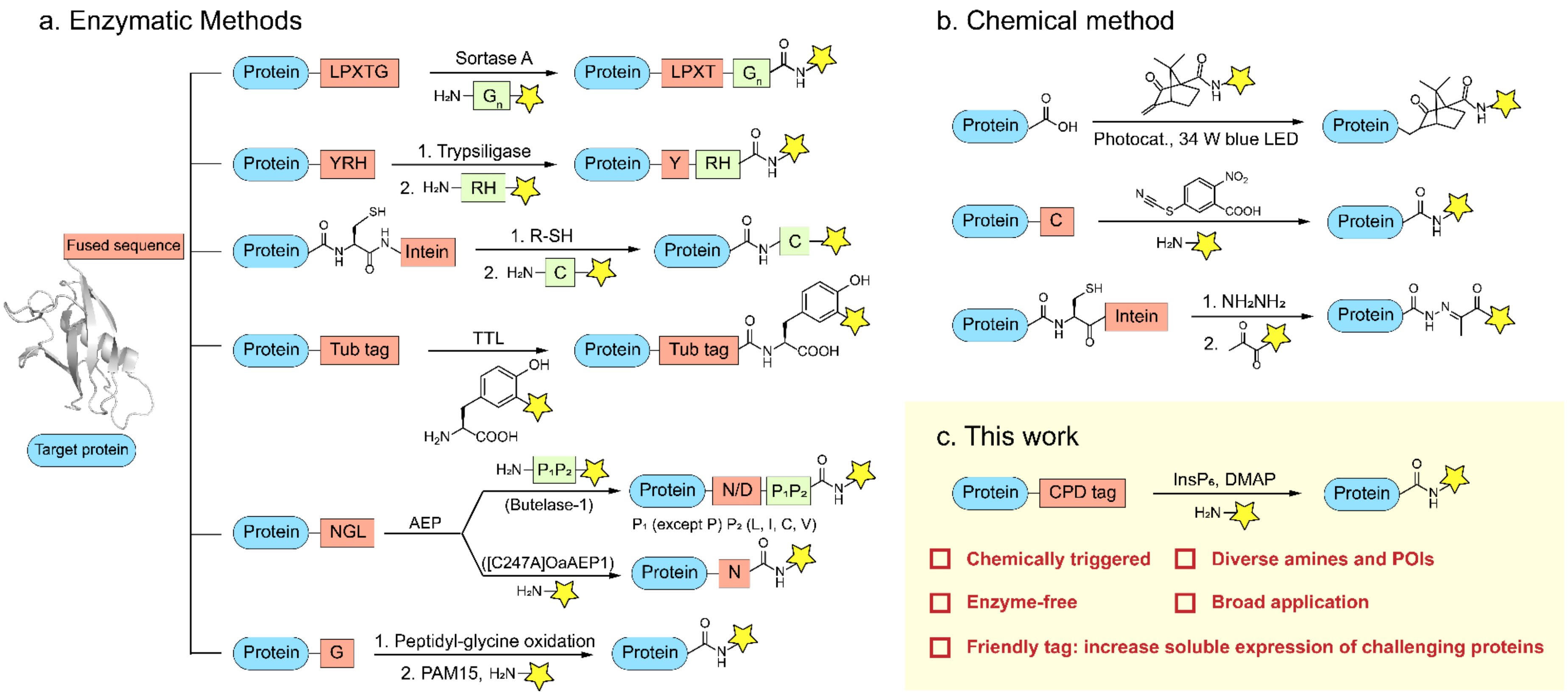

Supplementary Figure 1. Protein C-terminal functionalization strategies.

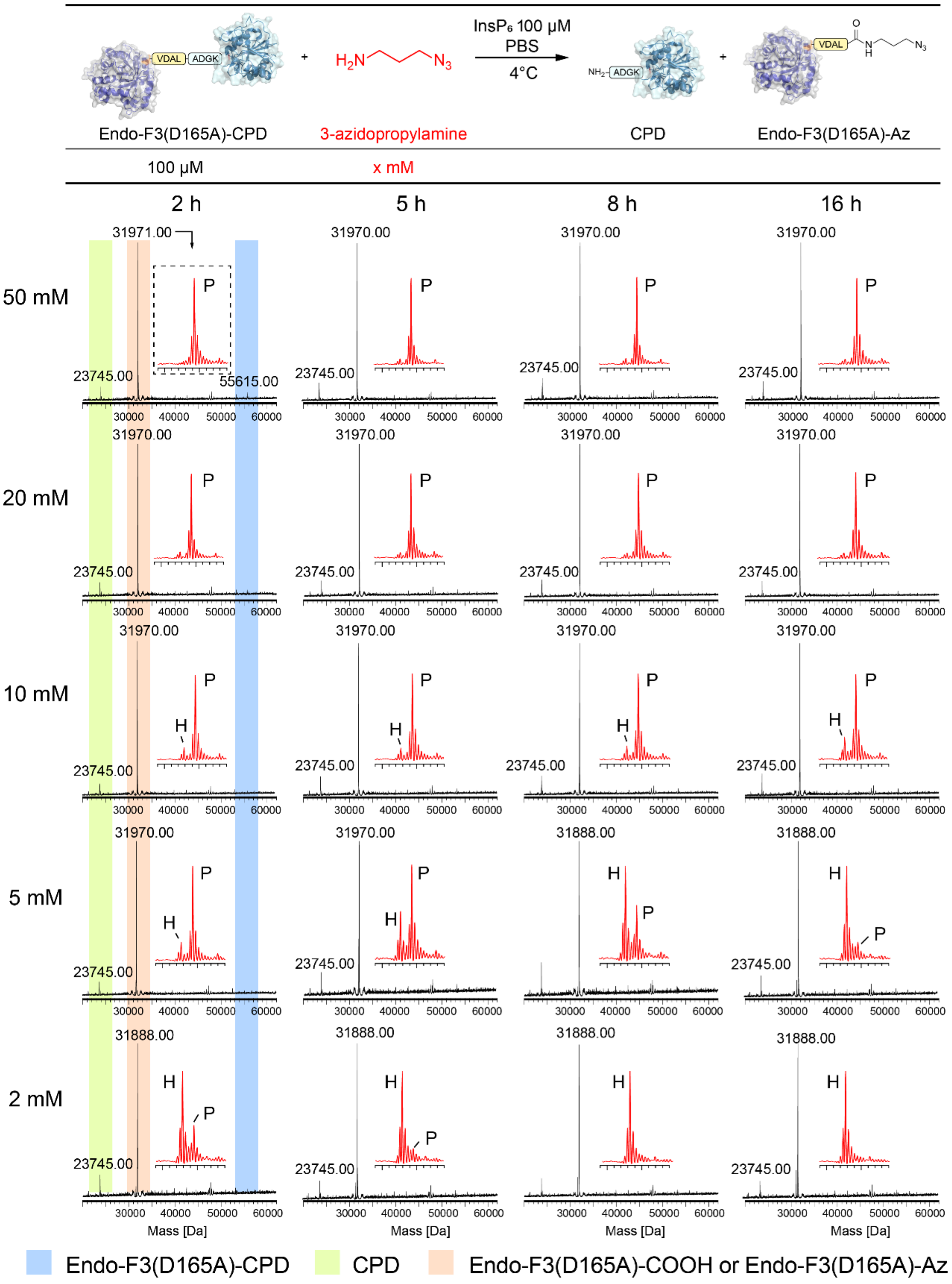

Supplementary Figure 2. MS spectra of CPD-mediated C-terminal azidation of Endo-F3(D165A) with different concentration of 3-azidepropylamine. Labeling reactions were conducted in PBS buffer containing CPD-tagged Endo-F3(D165A) at 100 μM, 3-azidepropylamine at 50 mM, 20 mM, 10 mM, 5 mM or 2 mM, and $InsP_6$ at 100 μM at 4°C for 16 h. The reaction was analyzed by ESI-TOF-MS at different time points. P: product; H: hydrolyzed.

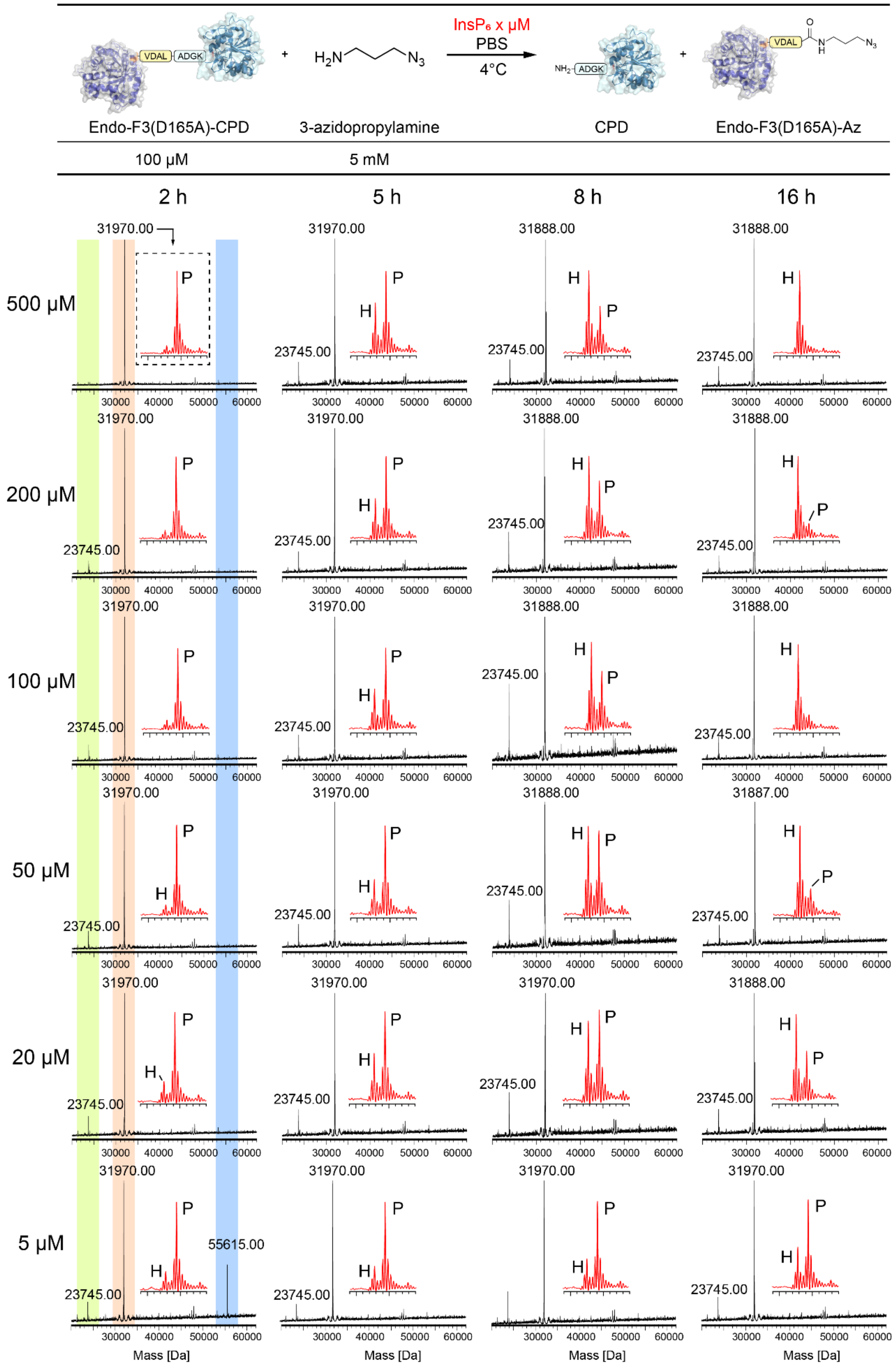
VDAL
ADGK
H_2N
N_3
$InsP_6$ x μM
PBS
4°C
NH_2
Endo-F3(D165A)-CPD
3-azidopropylamine
CPD
Endo-F3(D165A)-Az
100 μM
5 mM
2 h
5 h
8 h
16 h
500 μM
200 μM
100 μM
50 μM
20 μM
5 μM
31970.00
31888.00
31887.00
23745.00
55615.00
H
P
30000
40000
50000
60000
Mass [Da]

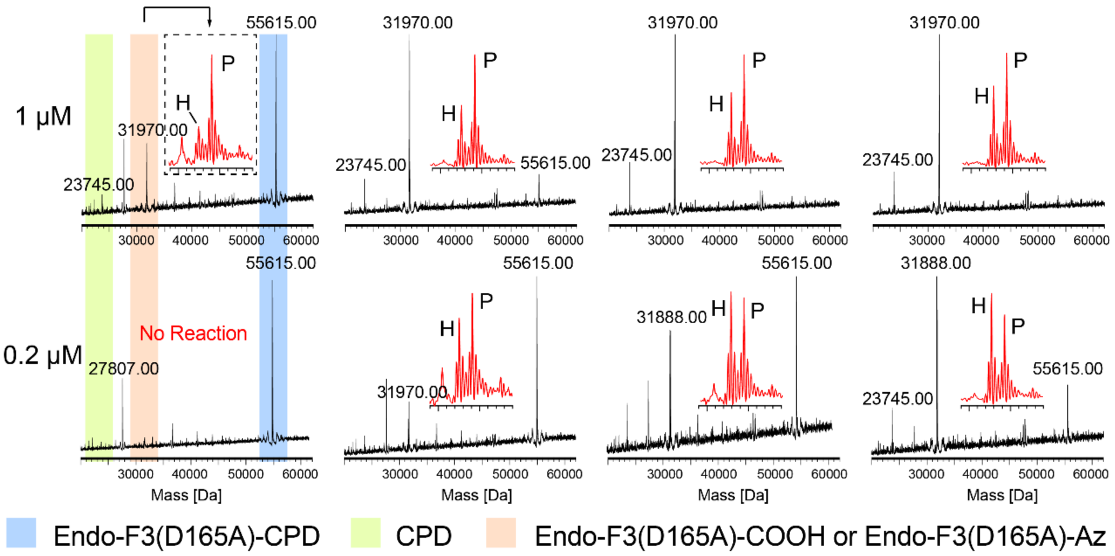

Supplementary Figure 3. MS spectra of CPD-mediated C-terminal azidation of Endo-F3(D165A) with different concentration of $InsP_6$. Labeling reactions were conducted in PBS buffer containing CPD-tagged Endo-F3(D165A) at 100 μM, 3-azidepropylamine at 5 mM, and $InsP_6$ at 500 μM, 200 μM, 100 μM, 50 μM, 20 μM, 5 μM, 1 μM or 0.2 μM at 4°C for 16 h. The reaction was analyzed by ESI-TOF-MS at different time points. P: product; H: hydrolyzed.

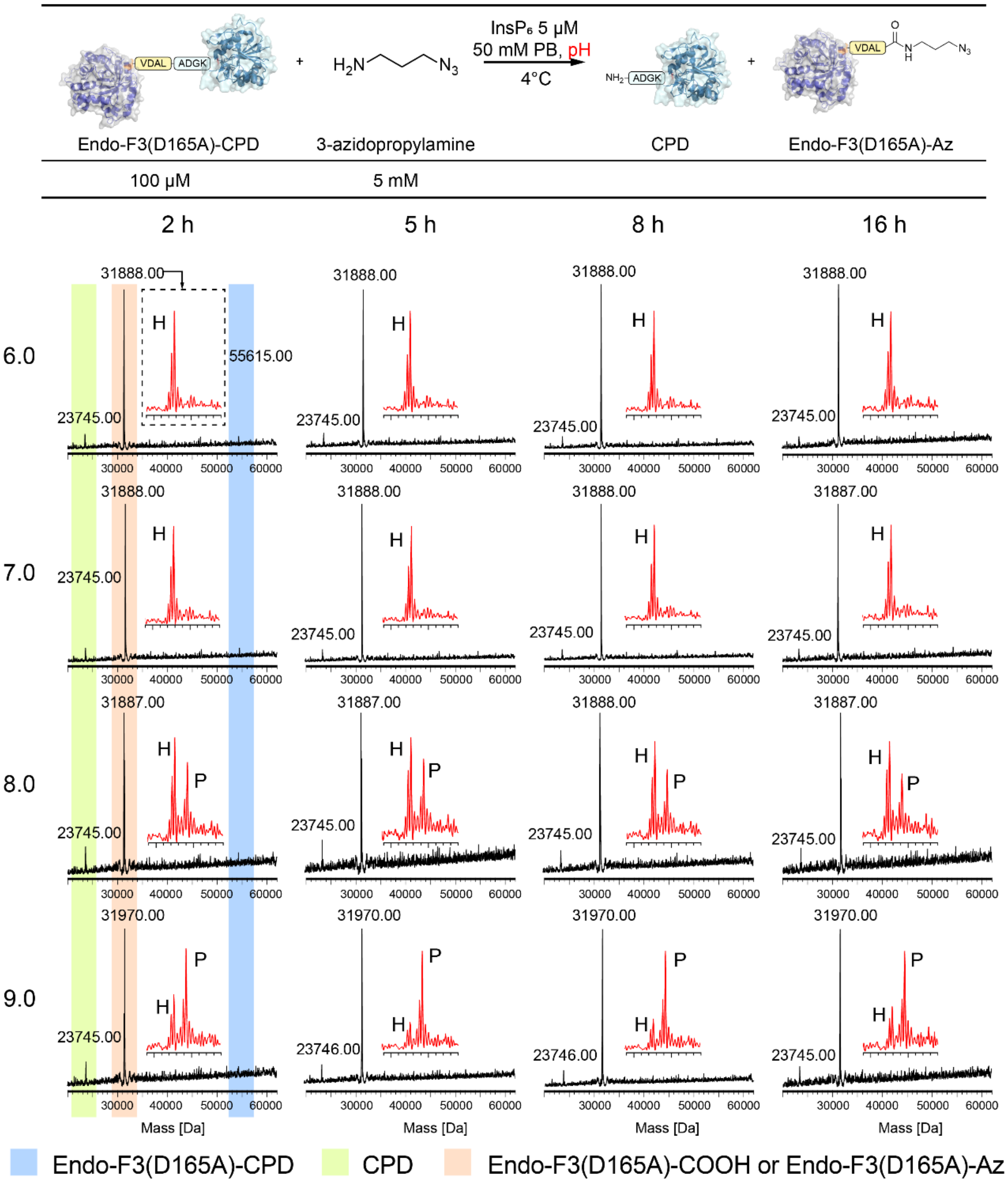

Supplementary Figure 4. MS spectra of CPD-mediated C-terminal azidation of Endo-F3(D165A) with different pH. Labeling reactions were conducted in 50 mM PB buffer (pH=6.0, 7.0, 8.0, 9.0), containing CPD-tagged Endo-F3(D165A) at 100 μM, 3-azidepropylamine at 5 mM, and $InsP_6$ at 5 μM at 4°C for 16 h. The reaction was analyzed by ESI-TOF-MS at different time points. P: product; H: hydrolyzed.

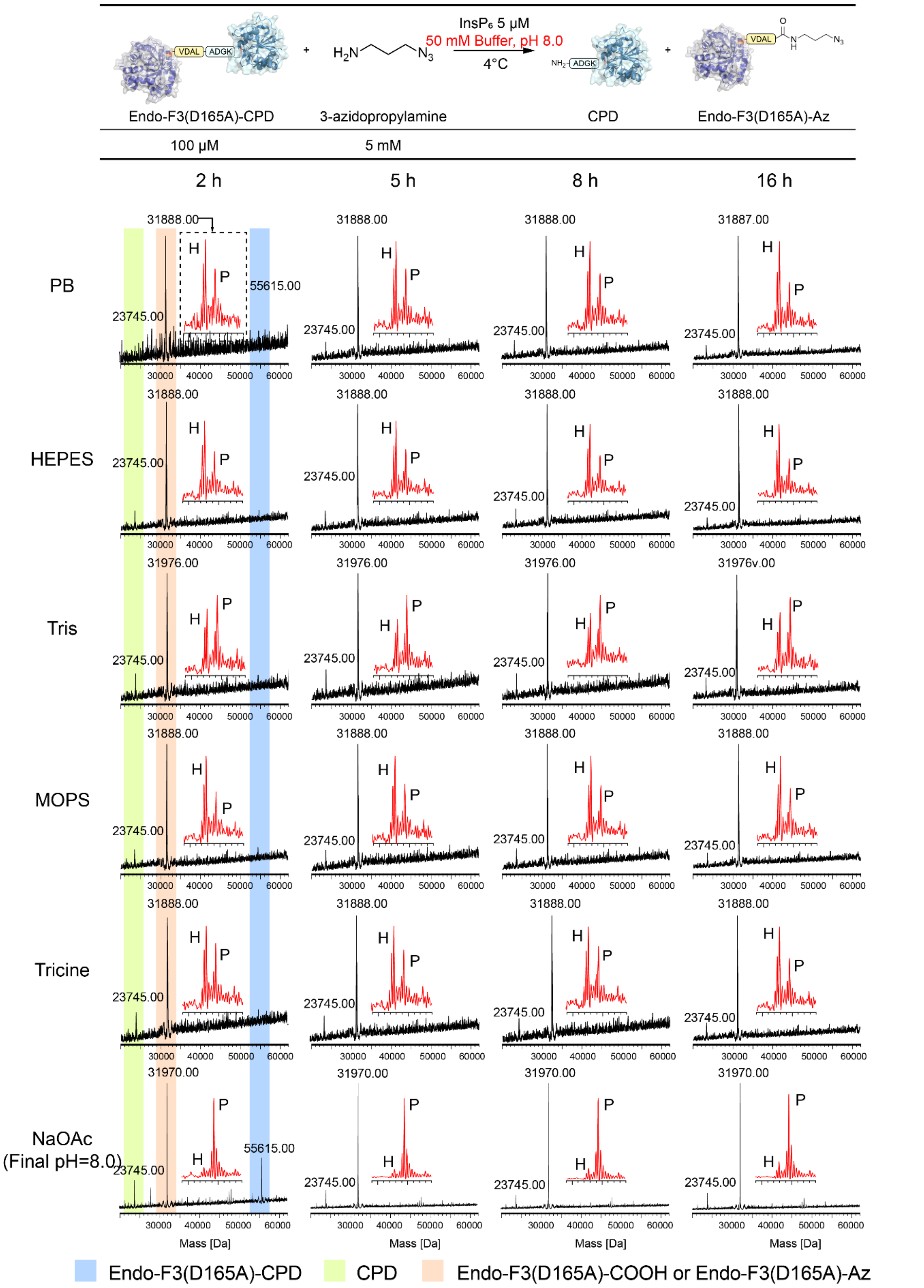

Supplementary Figure 5. MS spectra of CPD-mediated C-terminal azidation of Endo-F3(D165A) with different buffer. Labeling reactions were conducted in 50 mM PB, HEPES, Tris, MOPS, Tricine and NaOAc, containing CPD-tagged Endo-F3(D165A) at 100 μM, 3-azidepropylamine at 5 mM, and $InsP_6$ at 5 μM (Final pH=8.0) at 4°C for 16 h. The reaction was analyzed by ESI-TOF-MS at different time points. P: product; H: hydrolyzed.

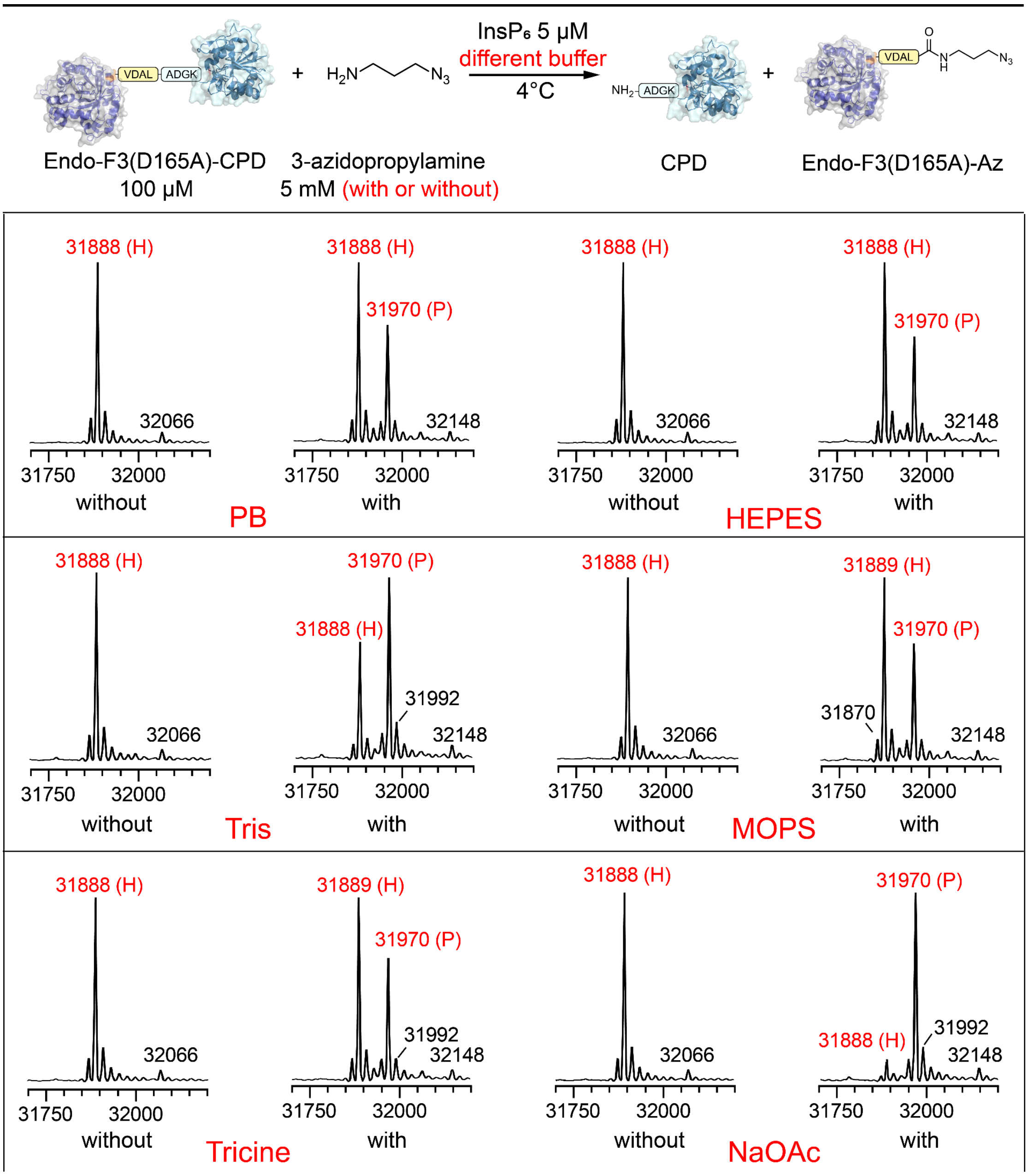

Supplementary Figure 6. Validation of the effect of different buffer to CPD-mediated C-terminal azidation of Endo-F3(D165A). Labeling reactions were conducted in 50 mM PB, HEPES, Tris, MOPS, Tricine and NaOAc, containing CPD-tagged Endo-F3(D165A) at 100 µM, with or without 3-azidepropylamine at 5 mM, and $InsP_6$ at 5 µM (Final pH=8.0) at 4°C for 5 h. The reaction was analyzed by ESI-TOF-MS at different time points. P: product; H: hydrolyzed.

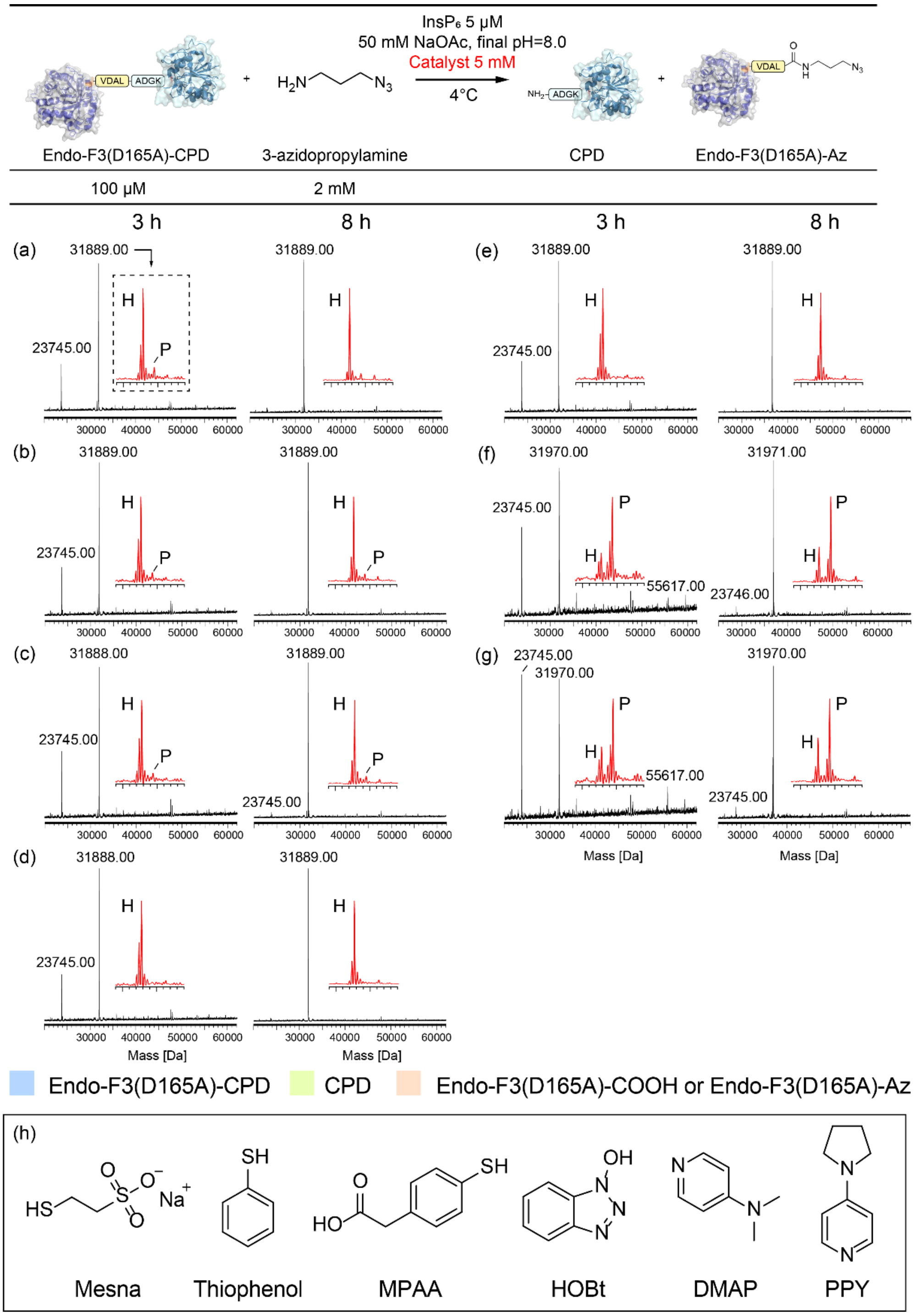

Supplementary Figure 7. MS spectra of CPD-mediated C-terminal azidation of Endo-F3(D165A) with different catalyst. Labeling reactions were conducted in NaOAc buffer (50 mM, final pH=8.0) containing CPD-tagged Endo-F3(D165A) at 100 μM, 3-azidepropylamine at 2 mM, $InsP_6$ at 5 μM, **(a)** without or with catalyst **(b)** Mesna; **(c)** Thiophenol; **(d)** 4-mercaptophenylacetic acid (MPAA); **(e)** N-Hydroxybenzotrizole (HOBt); **(f)** 4-Dimethylaminopyridine (DMAP); **(g)** 4-Pyrrolidinopyridine (PPY) at 4°C for 8 h. The reaction was analyzed by ESI-TOF-MS at different time points. **(h)** The structure of catalysts. P: product; H: hydrolyzed.

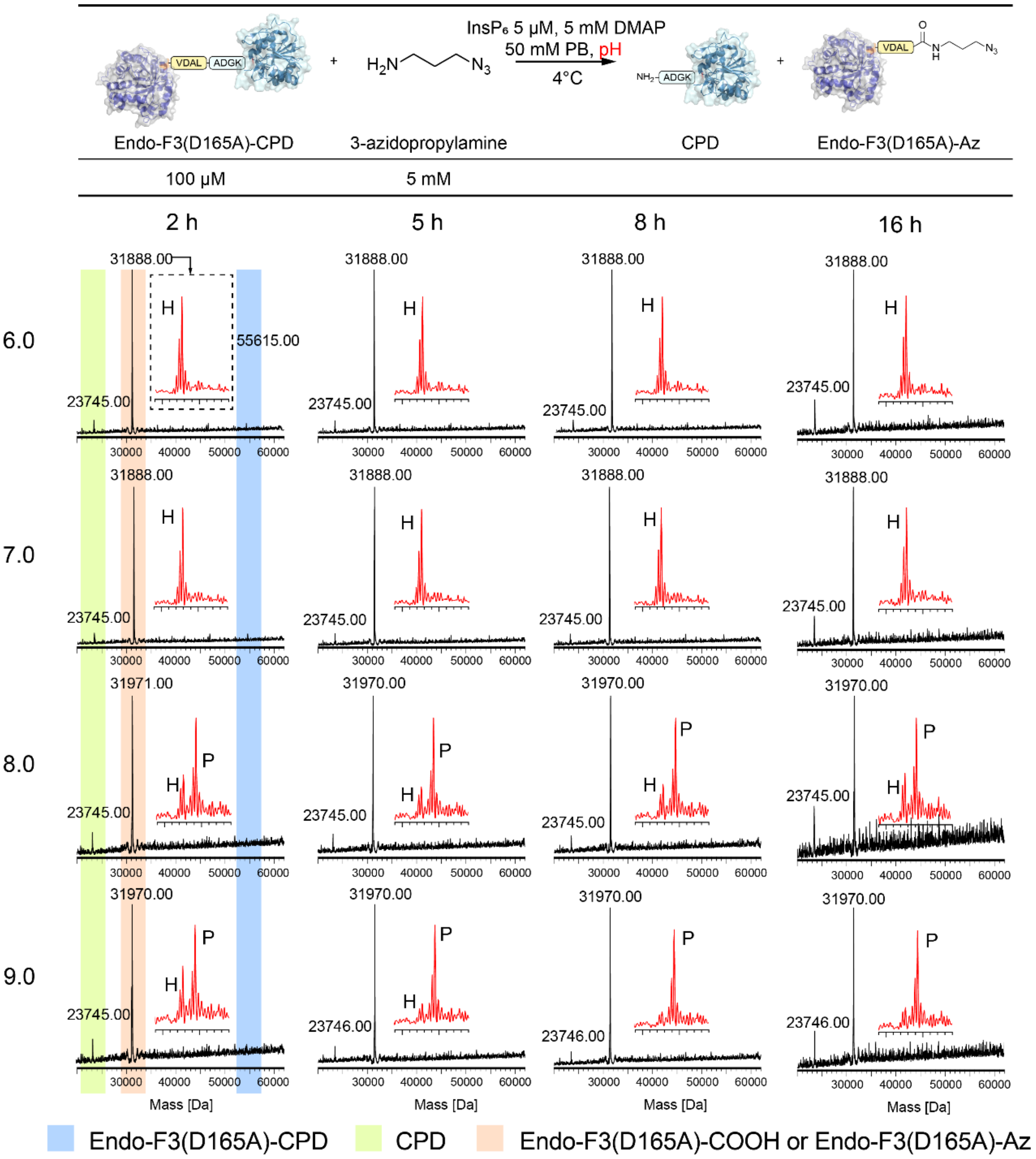

Supplementary Figure 8. MS spectra of CPD-mediated C-terminal azidation of Endo-F3(D165A) with DMAP and different pH. Labeling reactions were conducted in 50 mM PB buffer (pH=6.0, 7.0, 8.0, 9.0), containing CPD-tagged Endo-F3(D165A) at 100 μM, 3-azidepropylamine at 5 mM, DMAP at 5 mM and $InsP_6$ at 5 μM at 4°C for 16 h. The reaction was analyzed by ESI-TOF-MS at different time points. P: product; H: hydrolyzed.

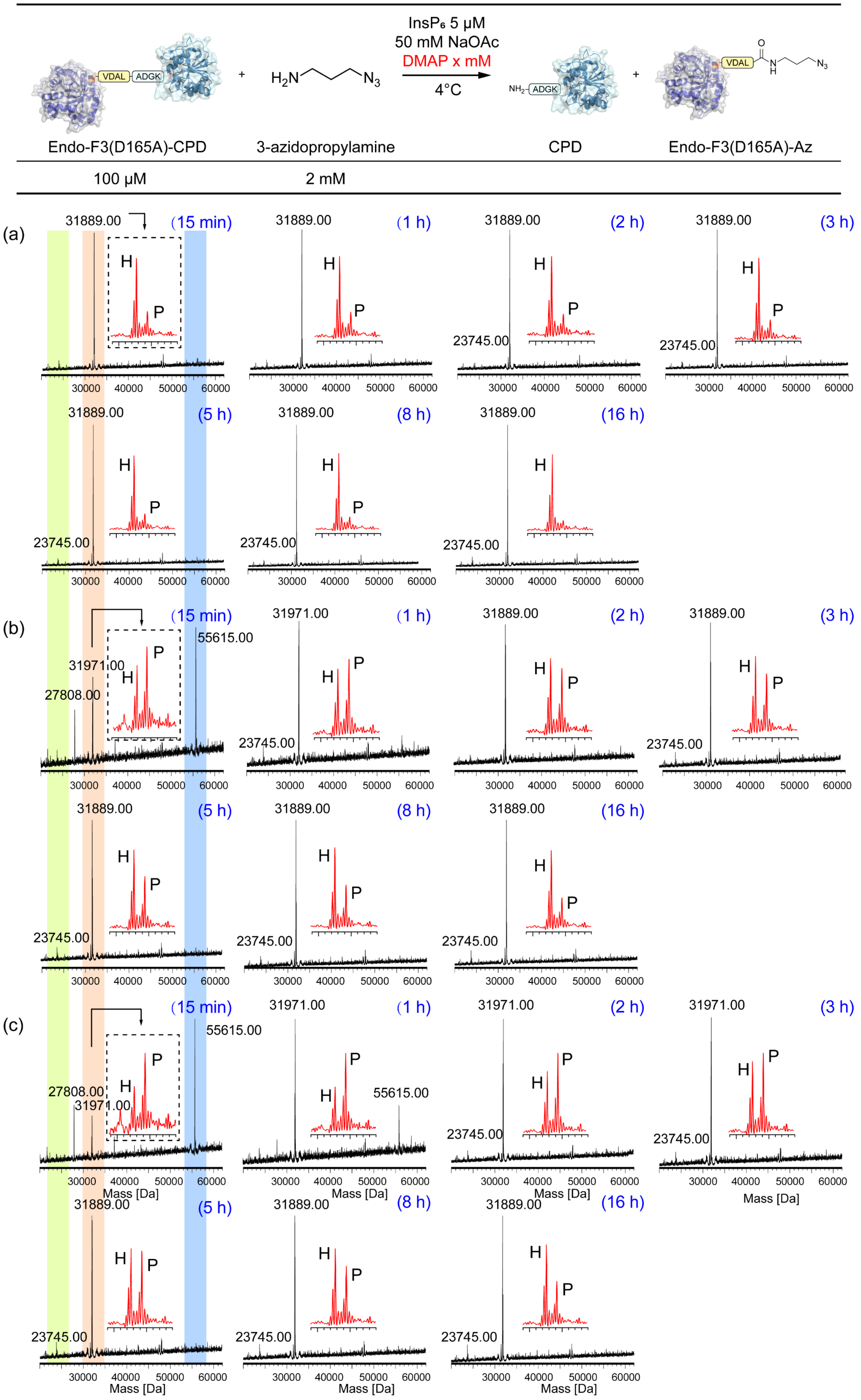

VDAL
ADGK
H_2N
N_3
InsP₆ 5 µM
50 mM NaOAc
DMAP x mM
4°C
NH_2-ADGK
Endo-F3(D165A)-CPD
3-azidopropylamine
CPD
Endo-F3(D165A)-Az
100 µM
2 mM
(a)
(b)
(c)
(15 min)
(1 h)
(2 h)
(3 h)
(5 h)
(8 h)
(16 h)
31889.00
31971.00
23745.00
27808.00
55615.00
H
P
30000
40000
50000
60000
Mass [Da]

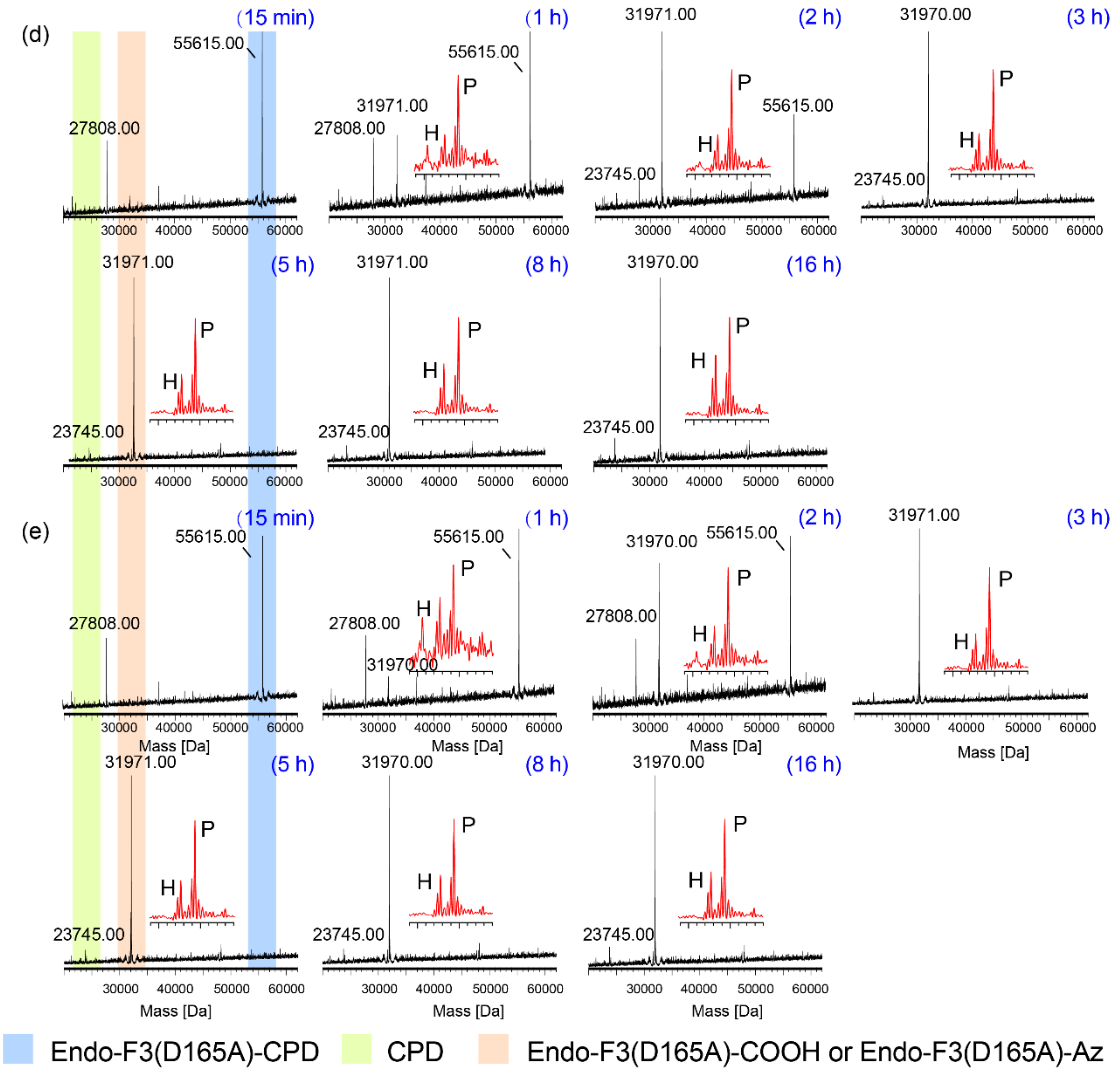

Supplementary Figure 9. MS spectra of CPD-mediated C-terminal azidation of Endo-F3(D165A) with different concentration of DMAP. Labeling reactions were conducted in NaOAc buffer (50 mM, Final pH=8.0) containing CPD-tagged Endo-F3(D165A) at 100 μM, 3-azidepropylamine at 2 mM, $InsP_6$ at 5 μM, DMAP at **(a)** 0 mM; **(b)** 1 mM; **(c)** 2 mM; **(d)** 5 mM; **(e)** 10 mM at 4°C for 16 h. The reaction was analyzed by ESI-TOF-MS at different time points. P: product; H: hydrolyzed.

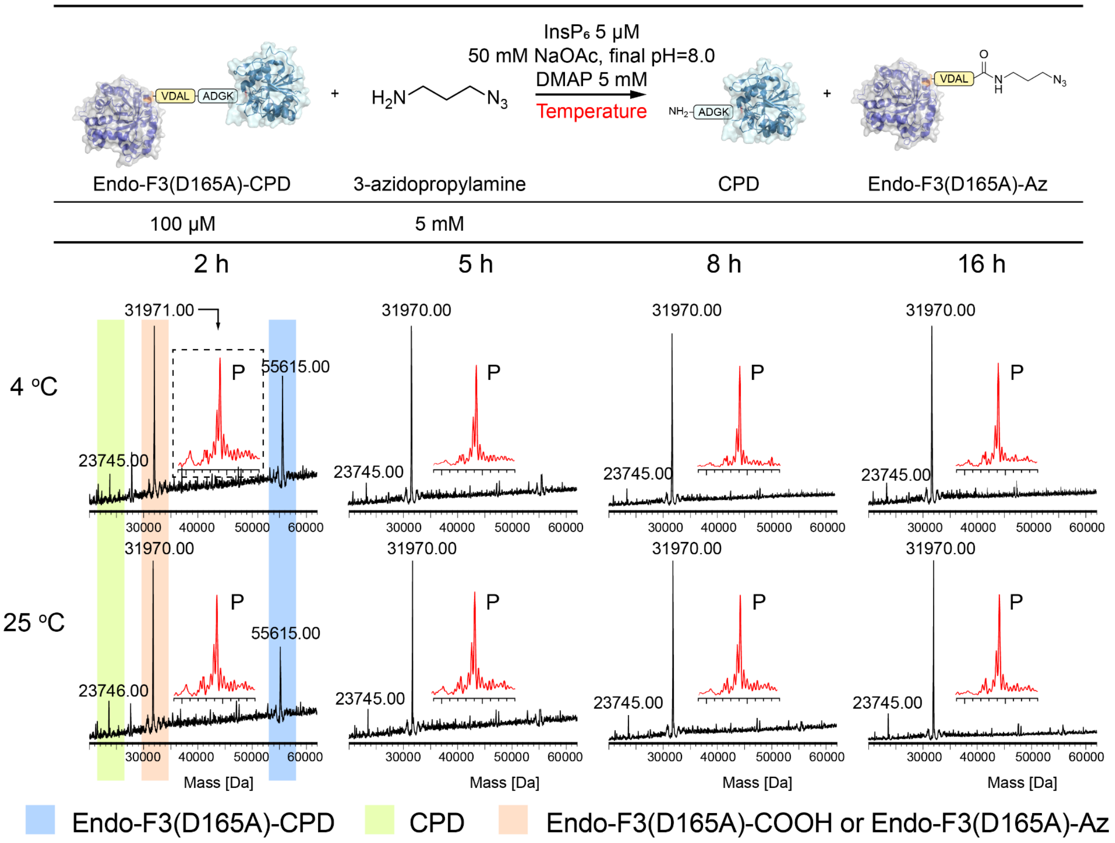

Supplementary Figure 10. MS spectra of CPD-mediated C-terminal azidation of Endo-F3(D165A) at different temperature. Labeling reactions were conducted in NaOAc buffer (50 mM, final pH=8.0) containing CPD-tagged Endo-F3(D165A) at 100 μM, 3-azidepropylamine at 5 mM, $InsP_6$ at 5 μM, DMAP at 5 mM at 4°C or 25°C for 16 h. The reaction was analyzed by ESI-TOF-MS at different time points. P: product; H: hydrolyzed.

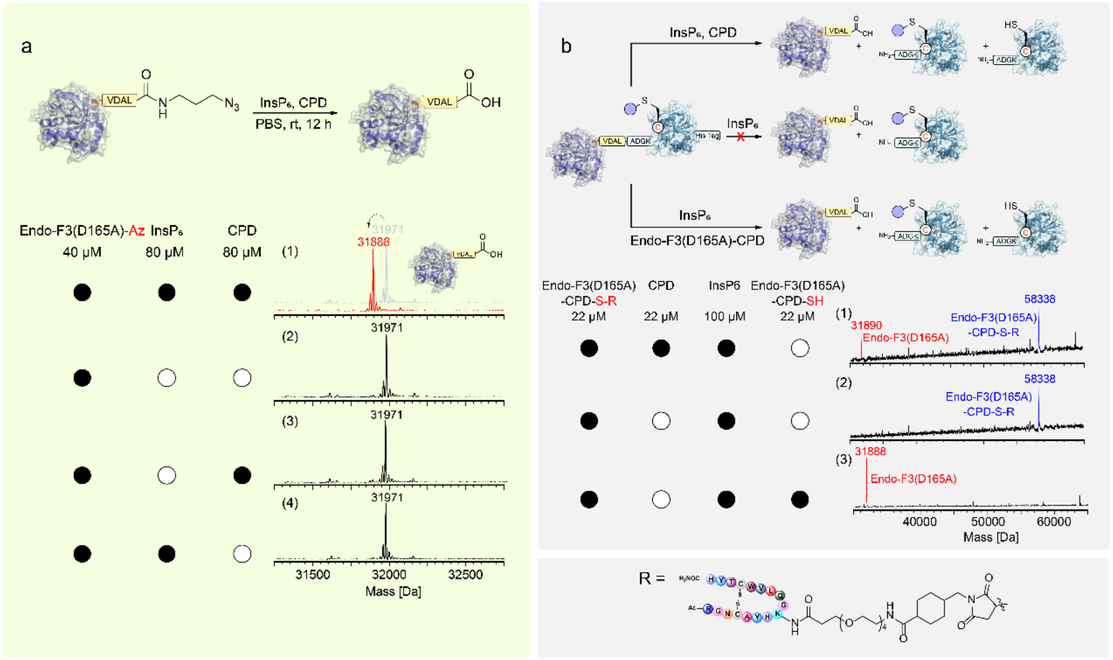

Supplementary Figure 11. Verification of further hydrolysis of C-terminal modification products in the presence of $InsP_6$ and CPD.

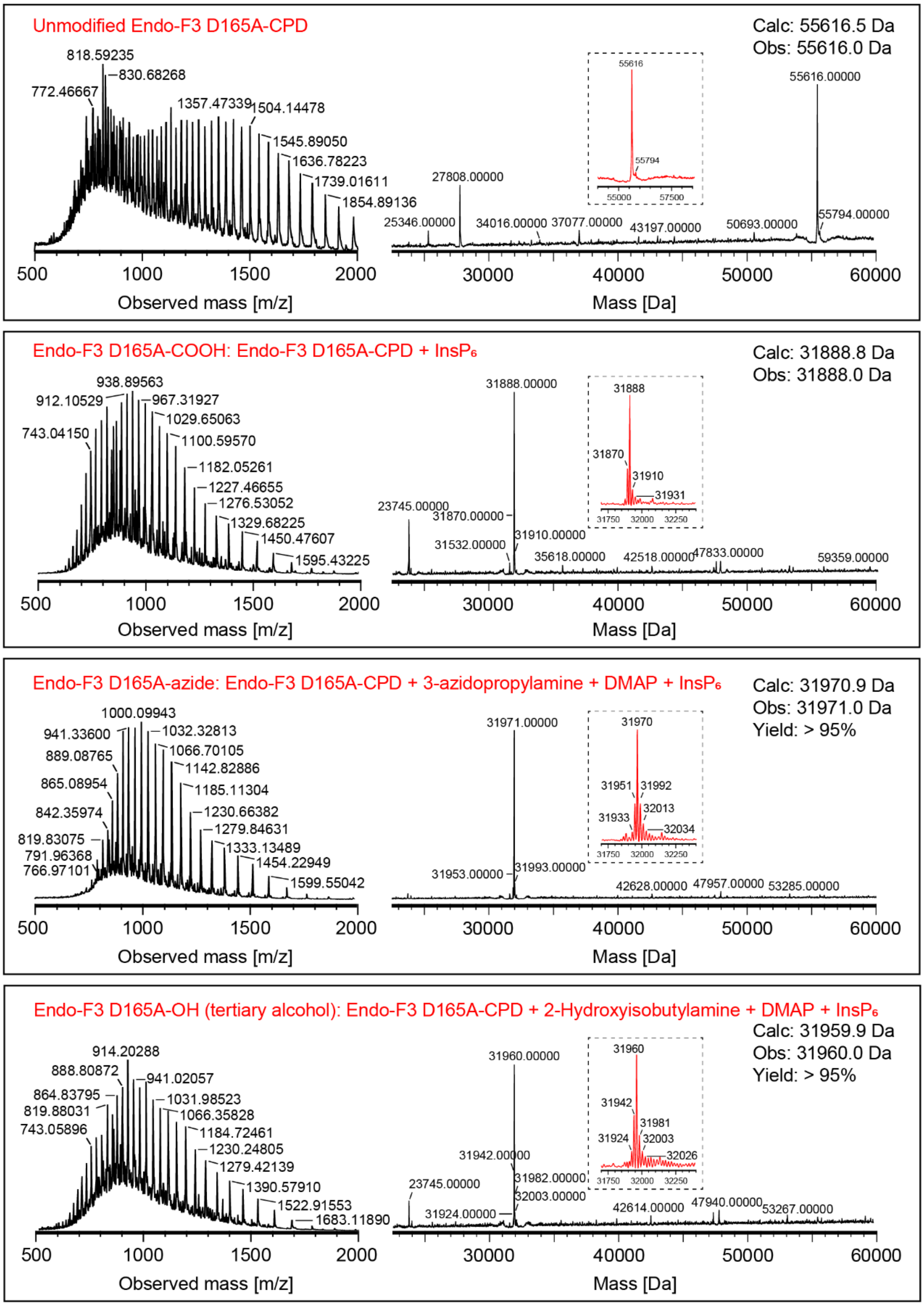

Supplementary Figure 12. MS spectra of Endo-F3(D165A) conjugates.

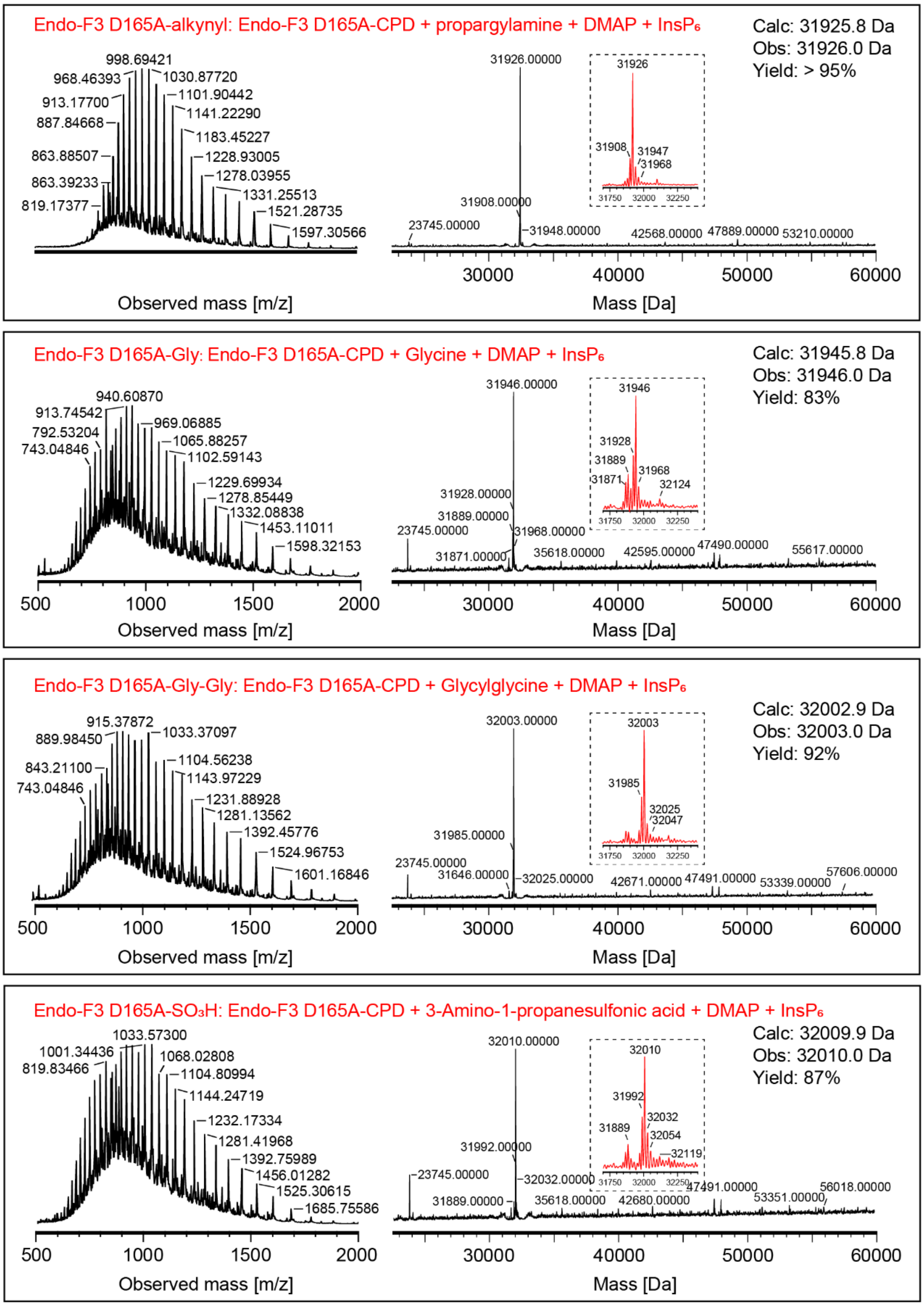

Supplementary Figure 12 (continue). MS spectra of Endo-F3(D165A) conjugates.

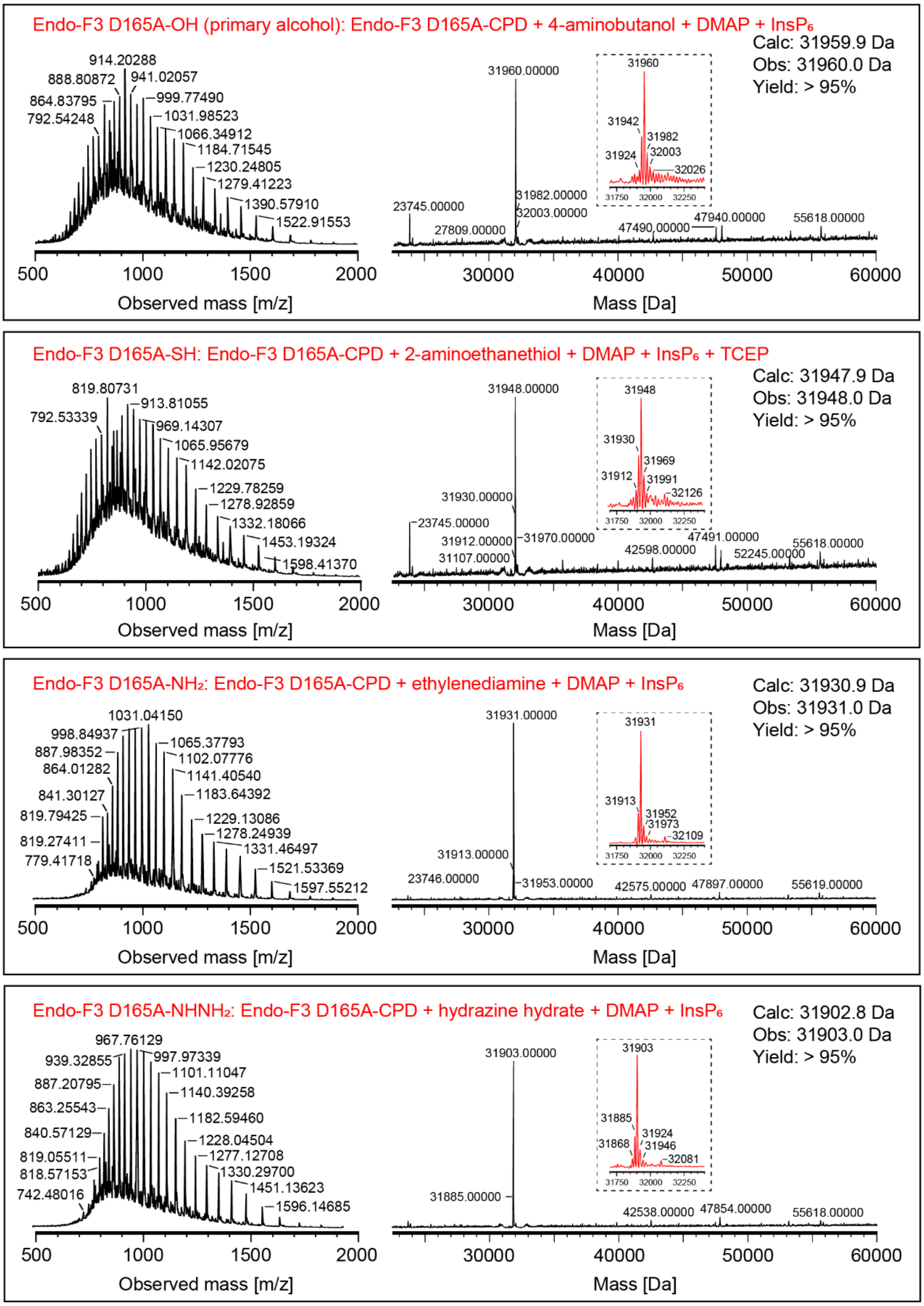

Supplementary Figure 12 (continue). MS spectra of Endo-F3(D165A) conjugates.

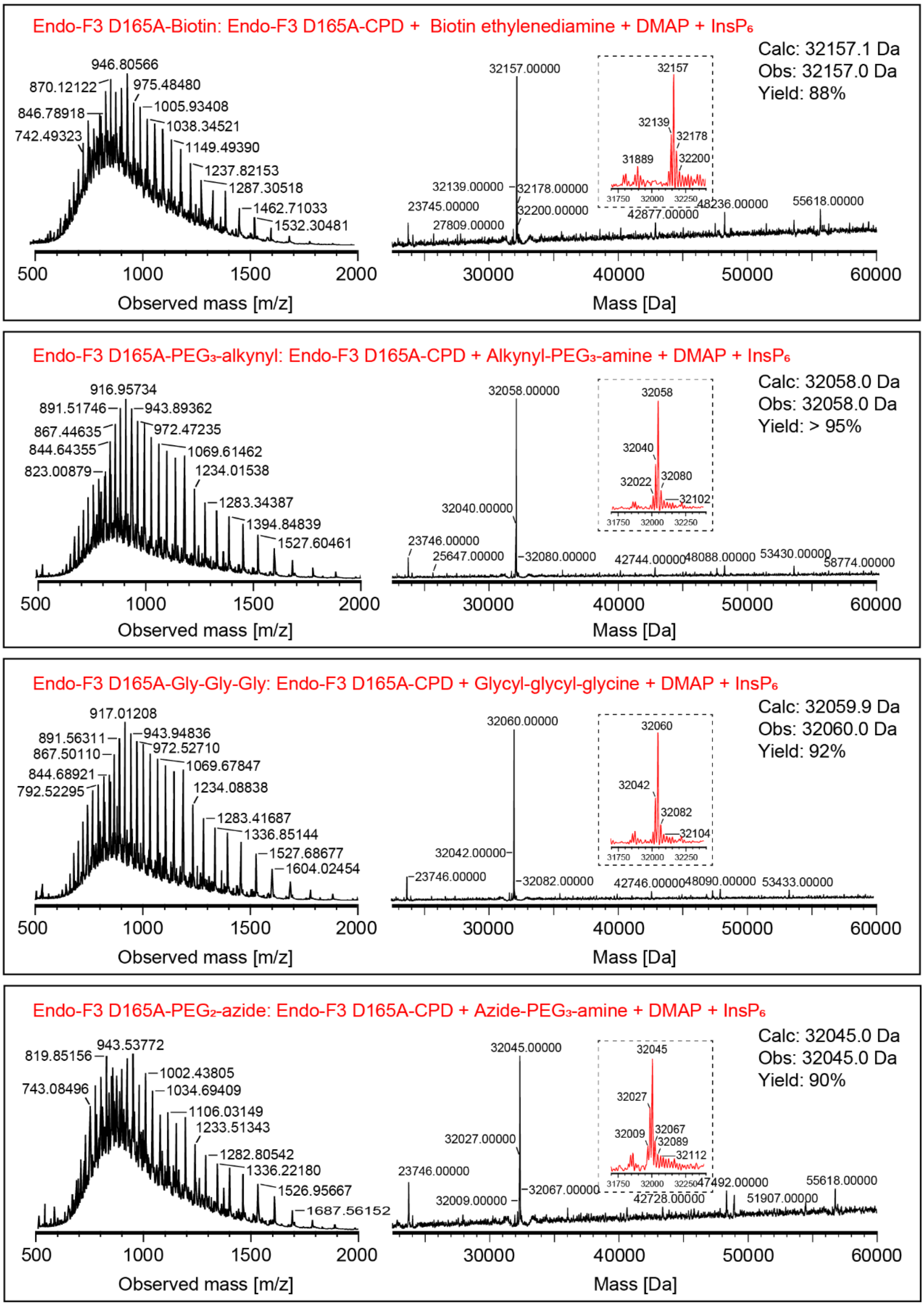

Supplementary Figure 12 (continue). MS spectra of Endo-F3(D165A) conjugates.

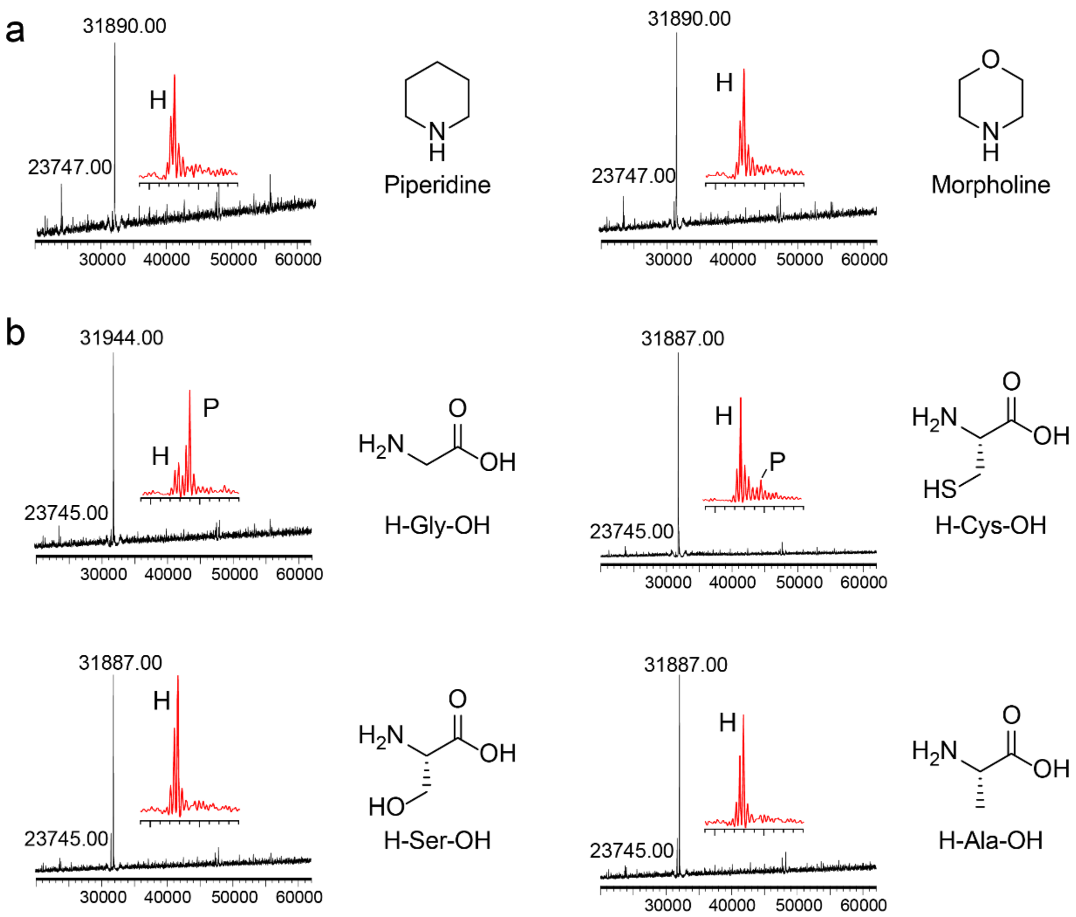

Supplementary Figure 13. CPD-mediated C-terminal modification of Endo-F3(D165A) with secondary-amine molecules (a) and amino acids (b) in the presence of $InsP_6$ and DMAP.

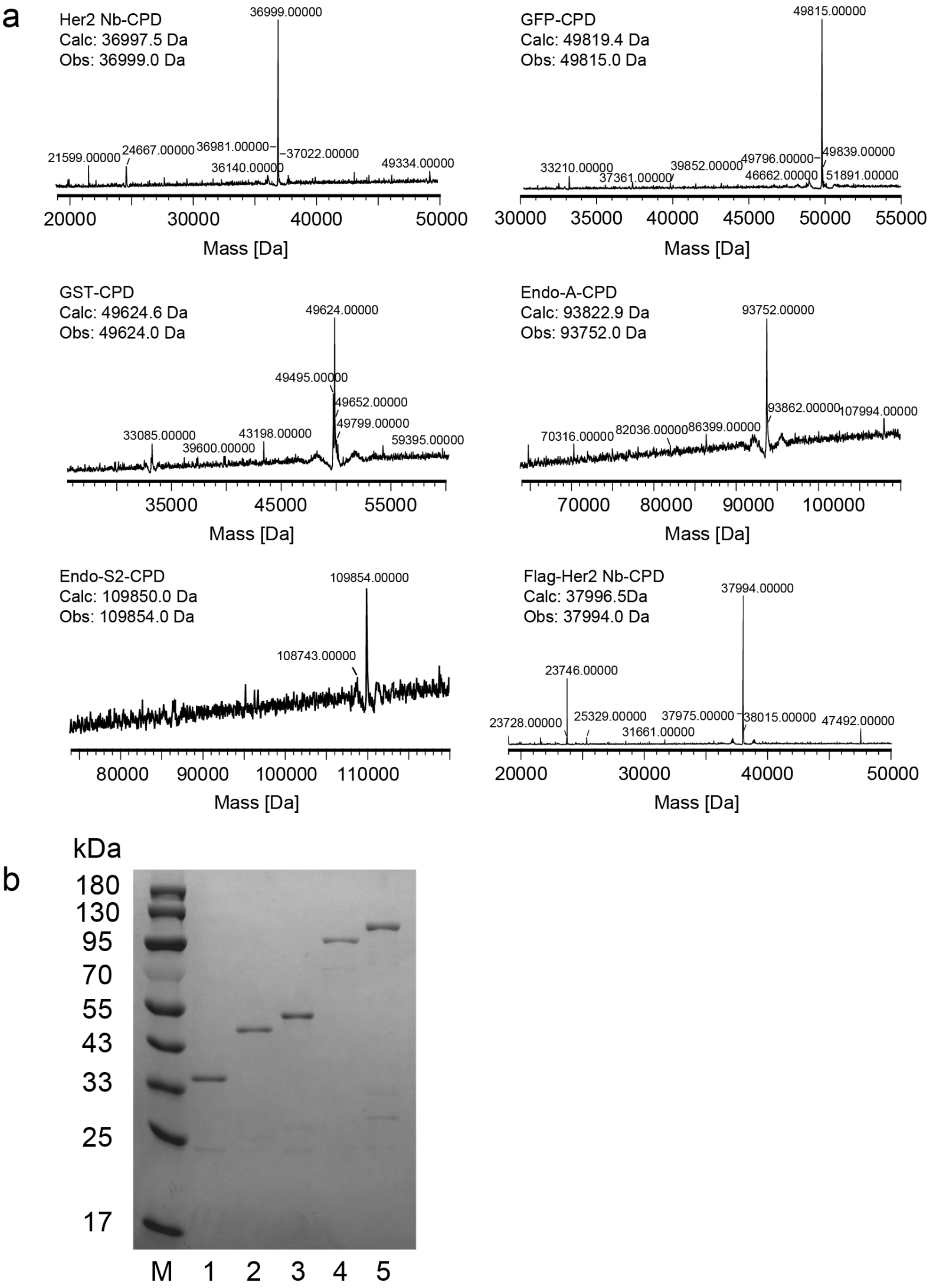

Supplementary Figure 14. MS spectra (a) and SDS-PAGE (b) profiles of POI-CPD conjugates. Lane M, molecular weight marker; lane 1, Her2 Nb-CPD; lane 2, GST-CPD; lane 3, GFP-CPD; lane 4, Endo-A-CPD; lane 5, Endo-S2-CPD;

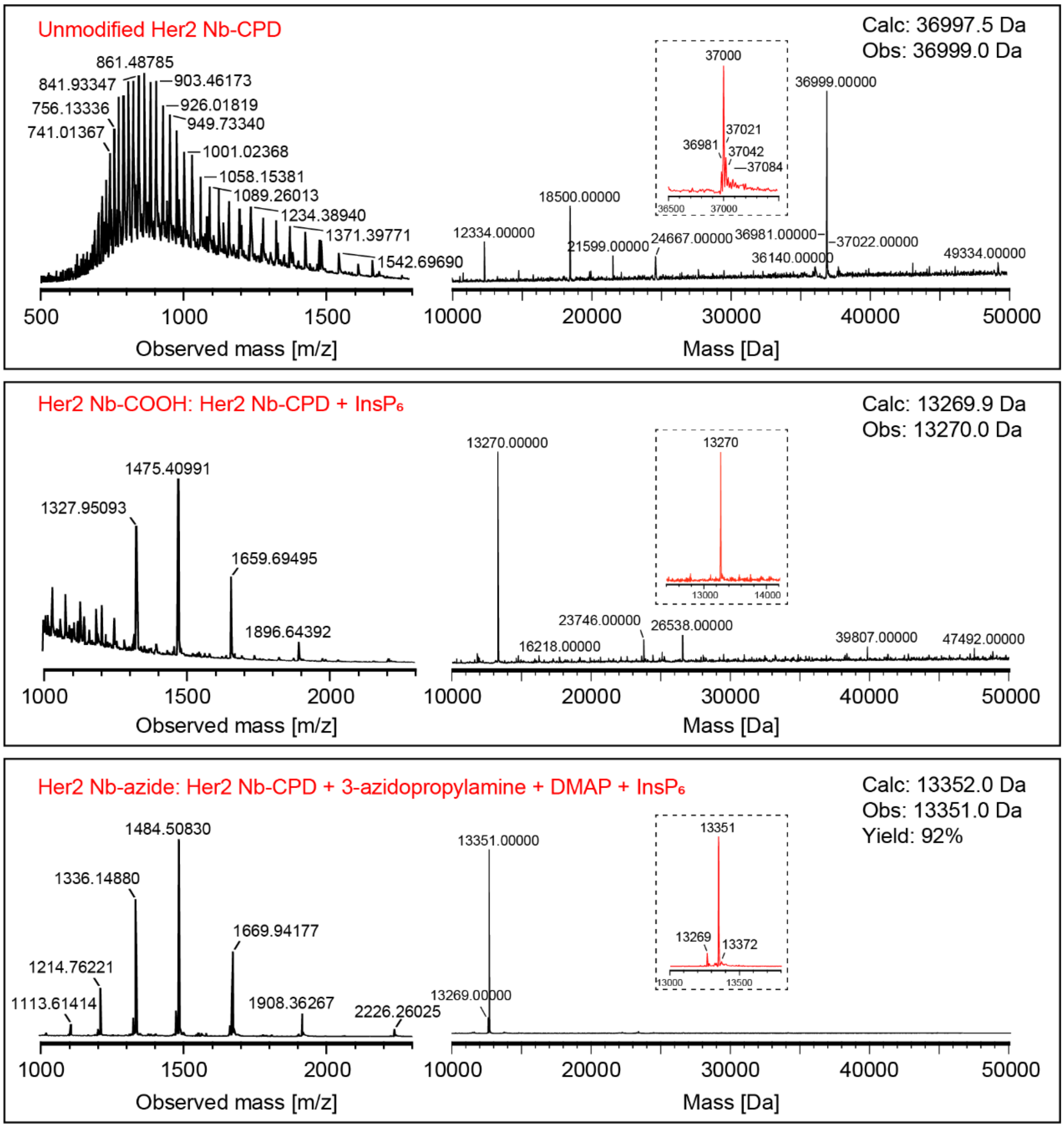

Supplementary Figure 15. MS spectra of Her2 Nanobody conjugates.

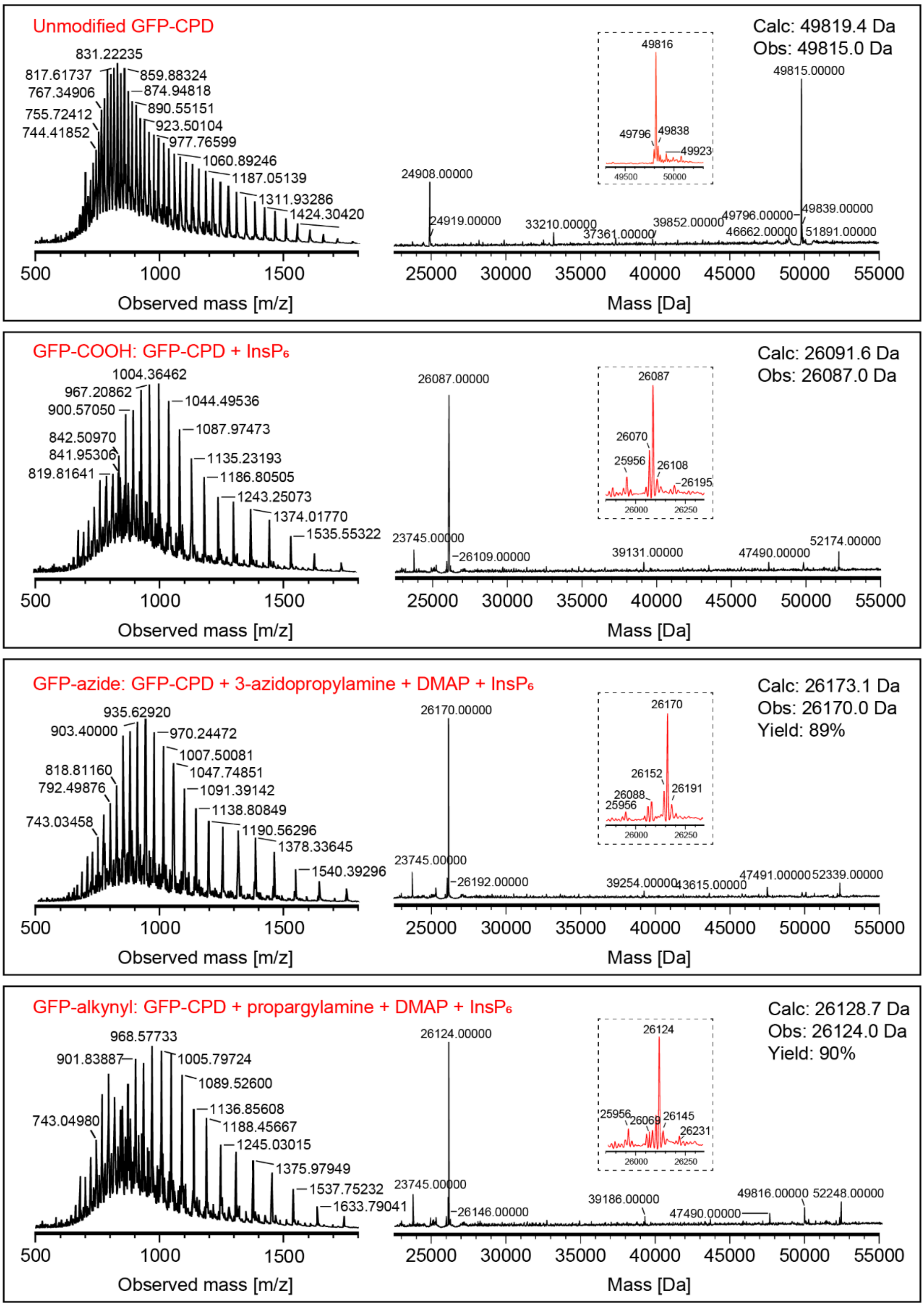

Supplementary Figure 16. MS spectra of GFP conjugates.

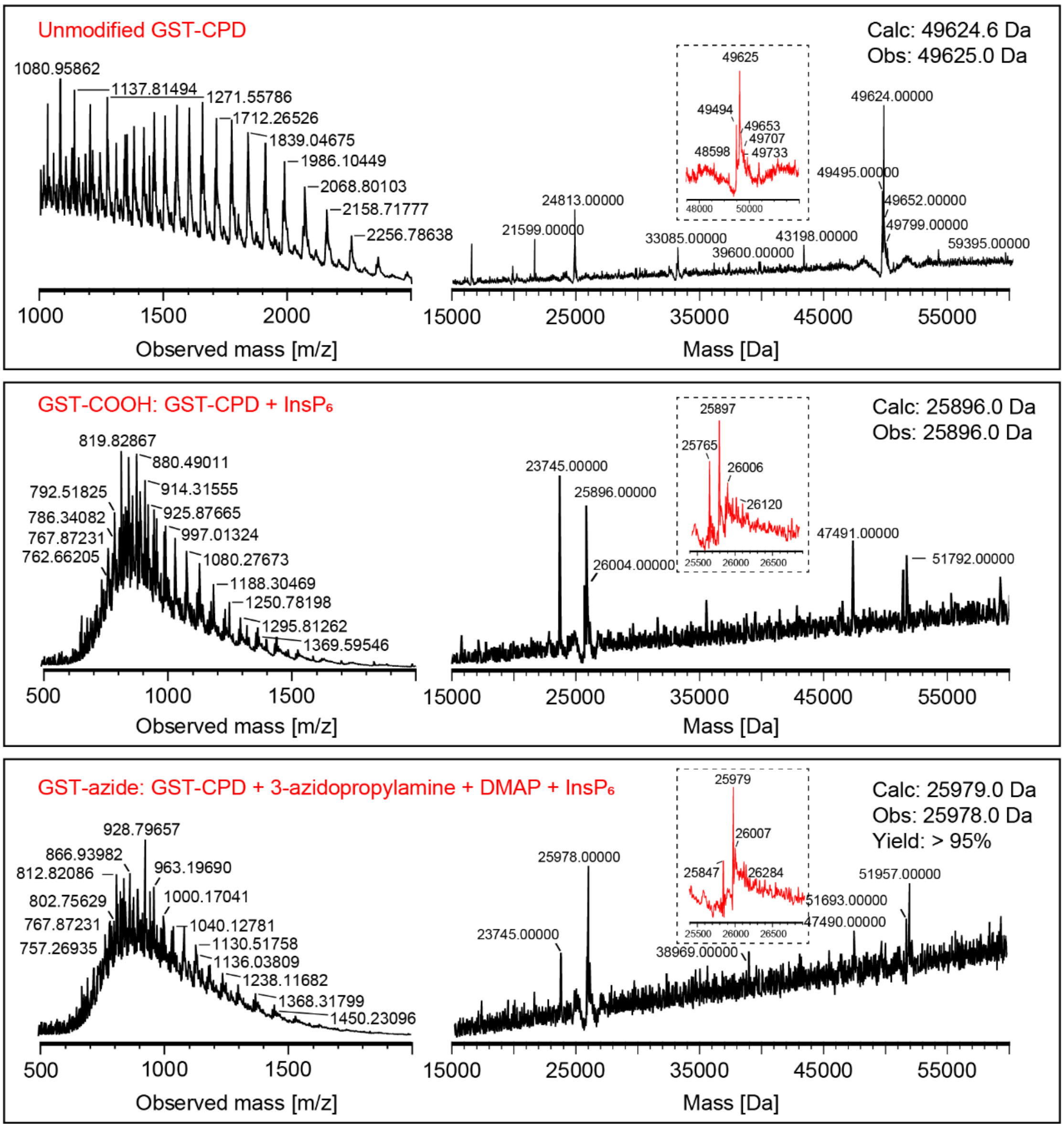

Supplementary Figure 17. MS spectra of GST conjugates.

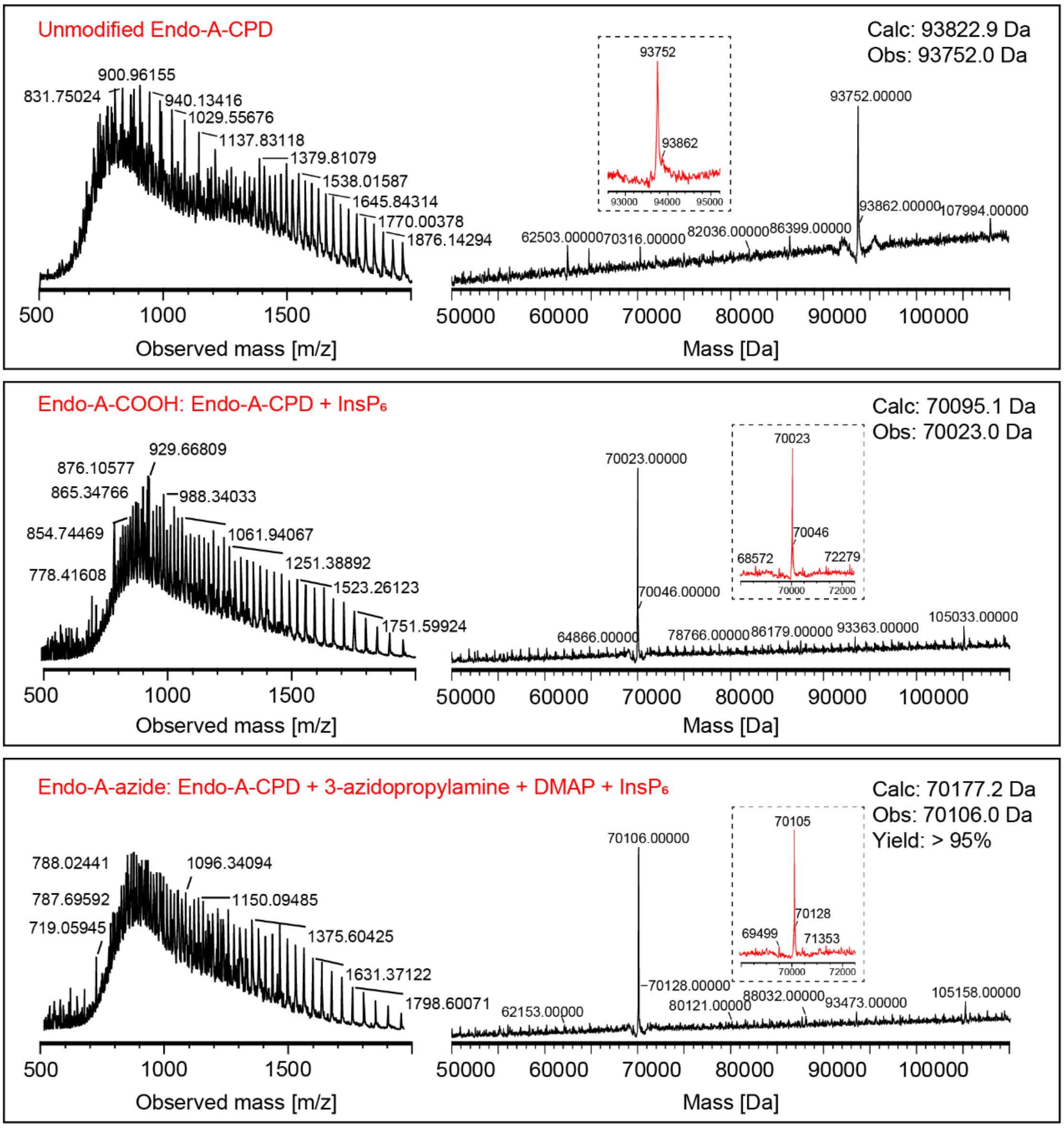

Supplementary Figure 18. MS spectra of Endo-A conjugates.

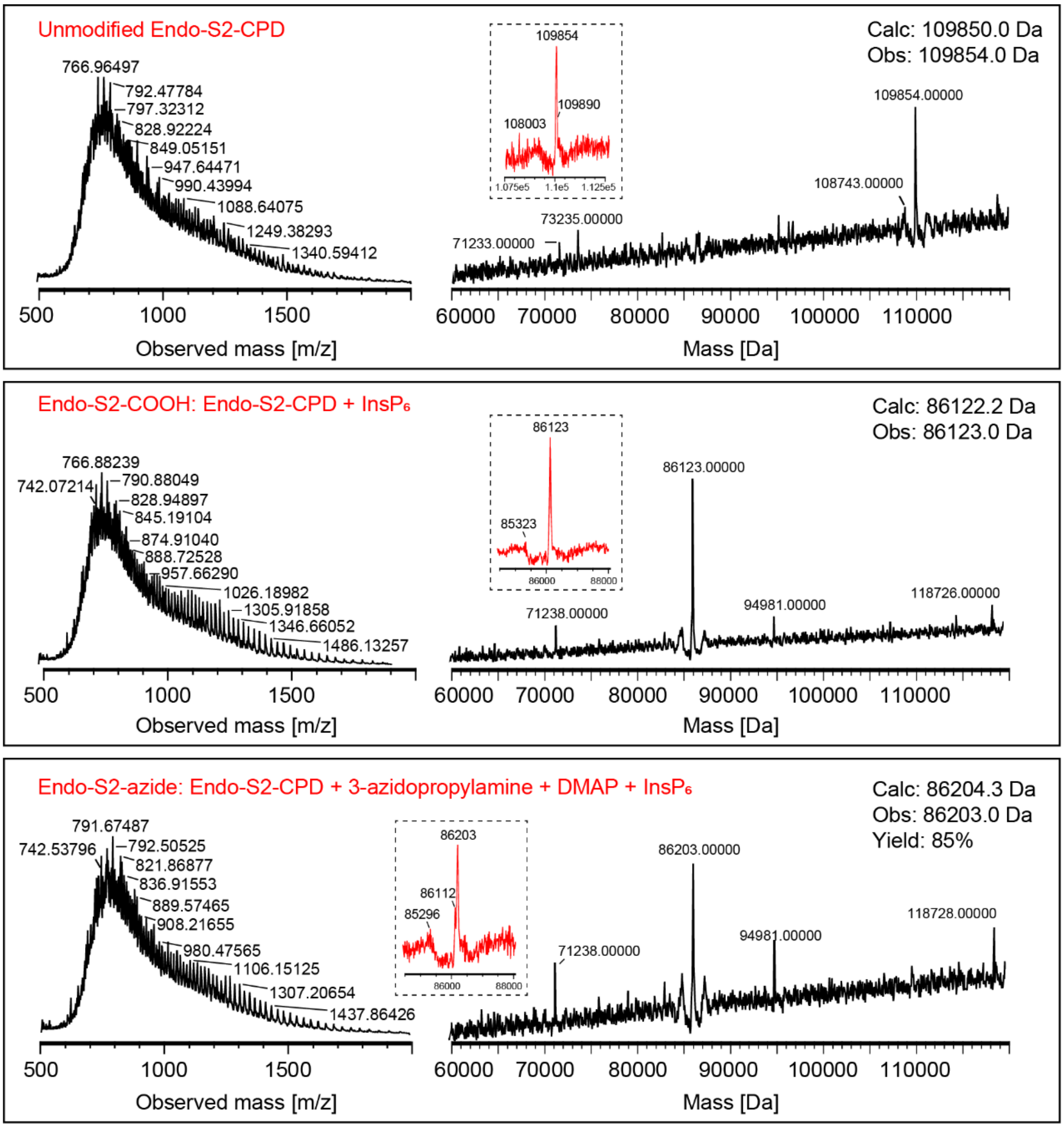

Supplementary Figure 19. MS spectra of Endo-S2 conjugates.

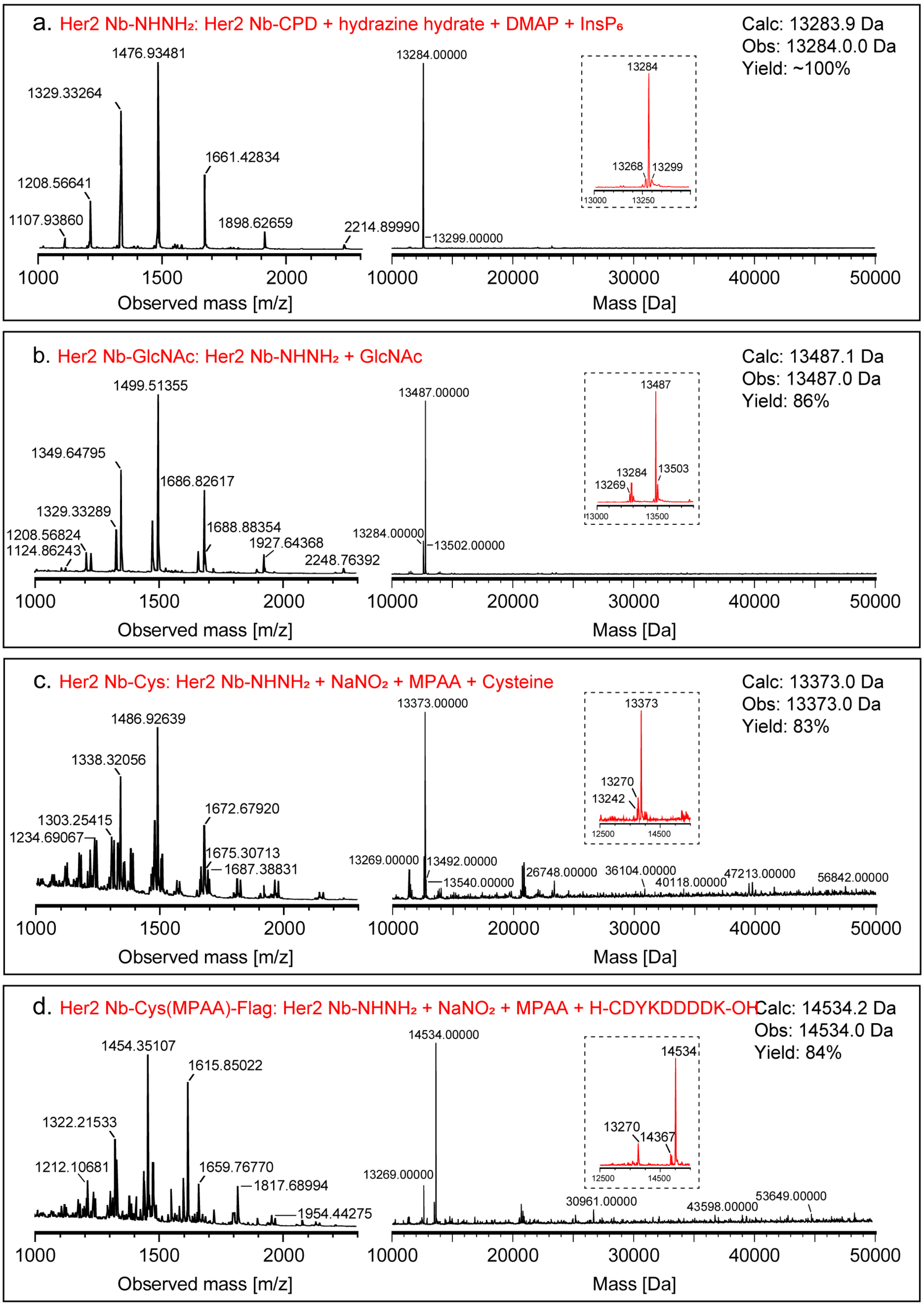

Supplementary Figure 20. MS spectra of Nb-NHNH$_2$ and its derivatization.

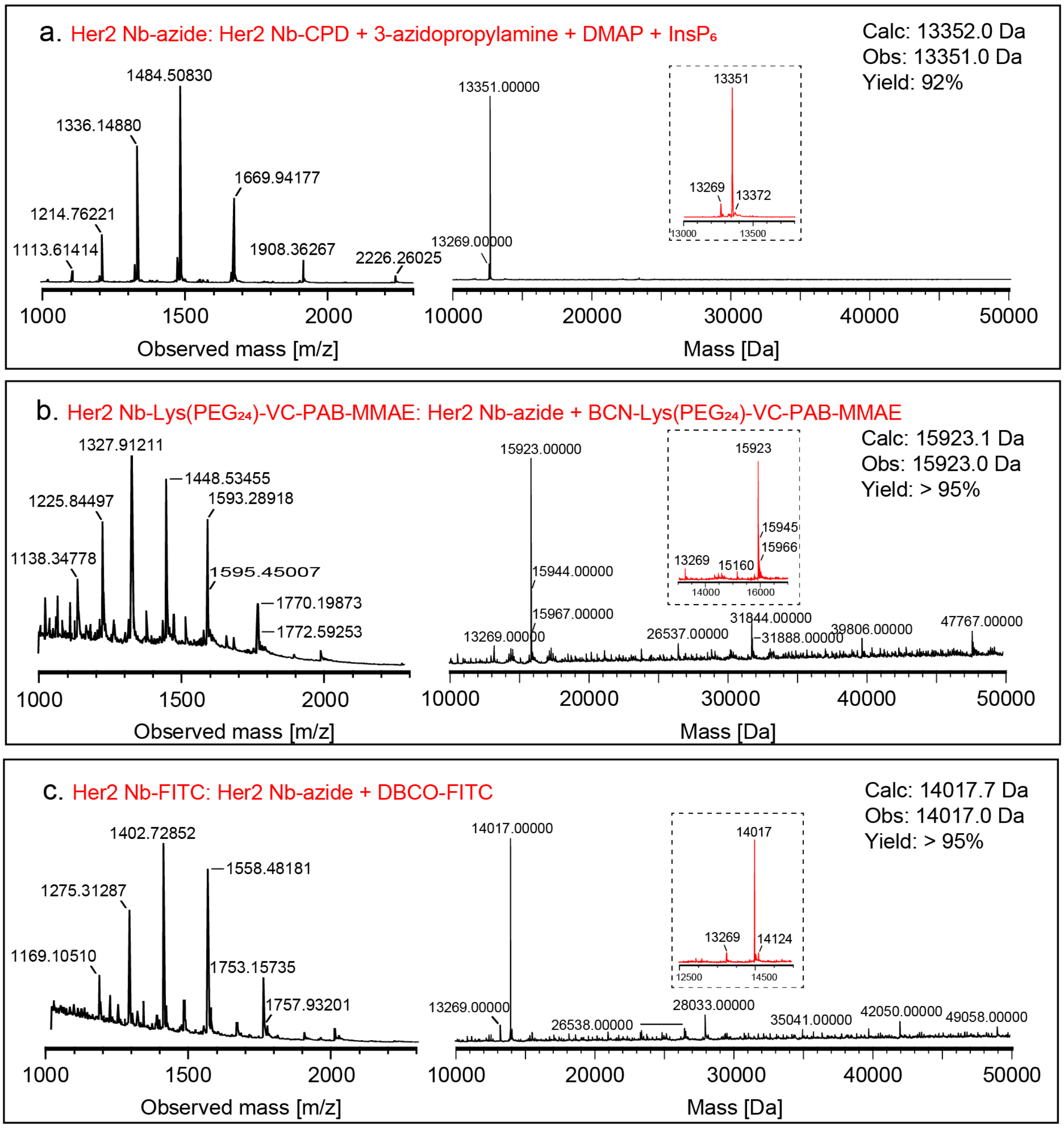

Supplementary Figure 21. MS spectra of Nb-azide and its derivatization.

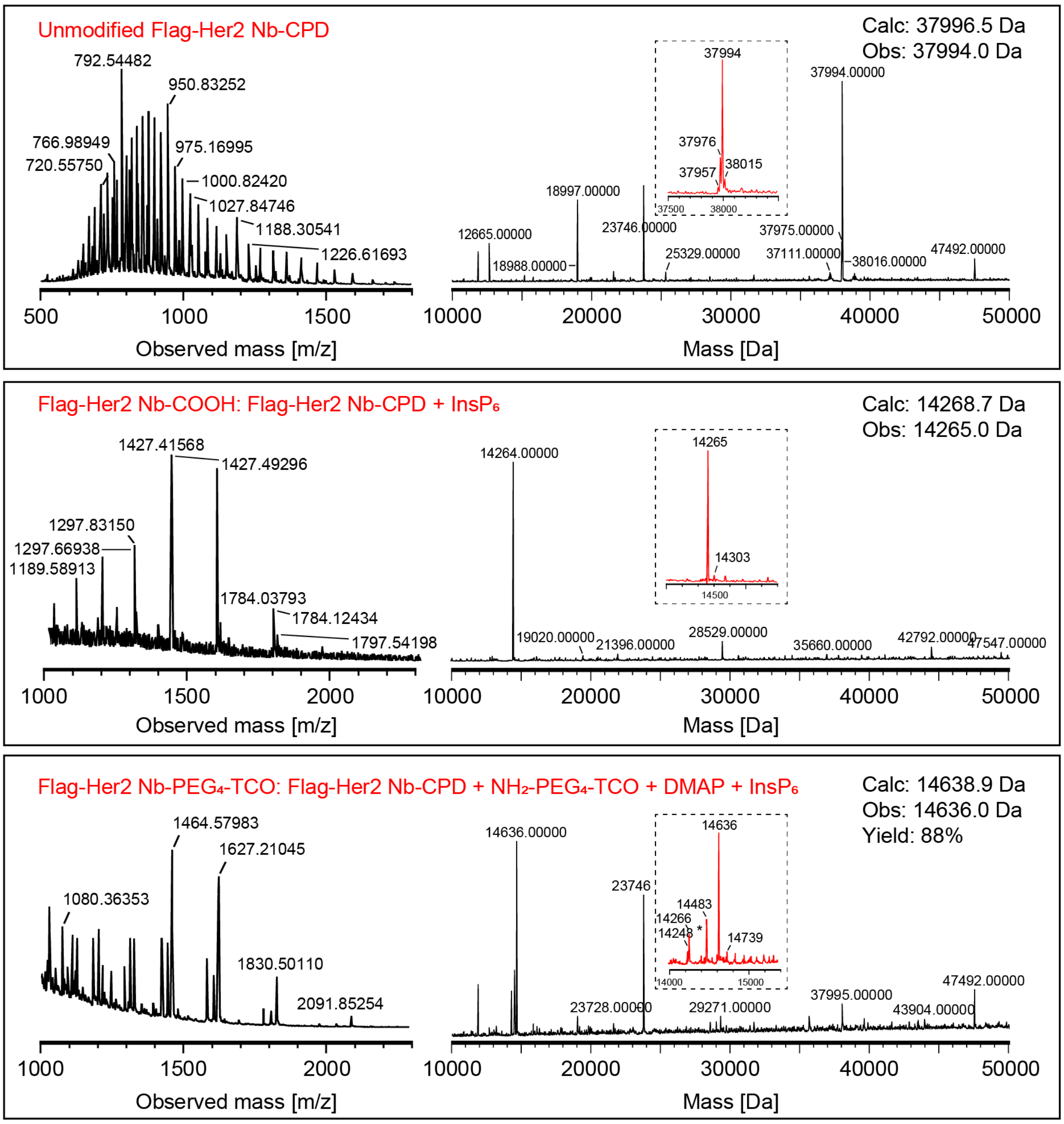

Supplementary Figure 22. MS spectra of Flag-Her2 Nb conjugates. *Mass fragments, the TCO was broken in mass.

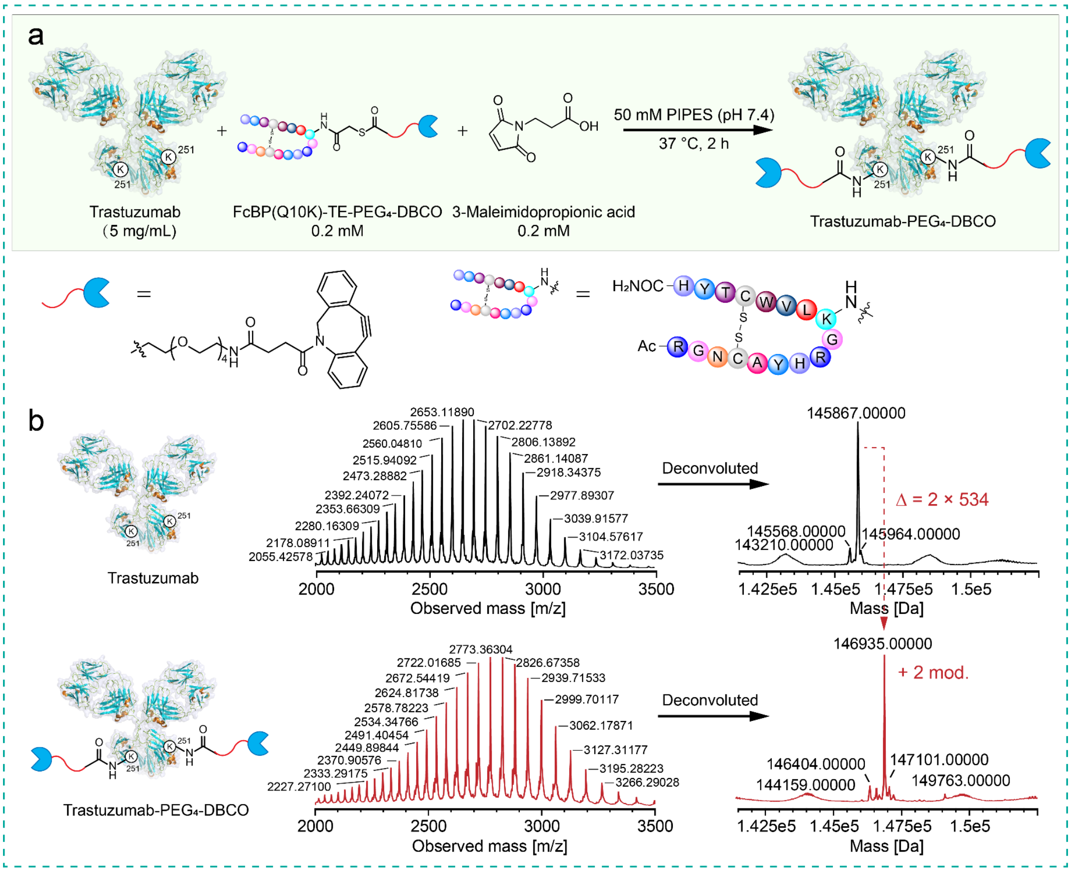

Supplementary Figure 23. Synthesis of DBCO-tagged Trastuzumab by FcBP(Q10K)-TE-PEG4-DBCO. **a.** Scheme of one-step synthesis of DBCO-tagged Trastuzumab. **b.** LC-MS profiles of Trastuzumab (upper) and DBCO-tagged Trastuzumab (lower).

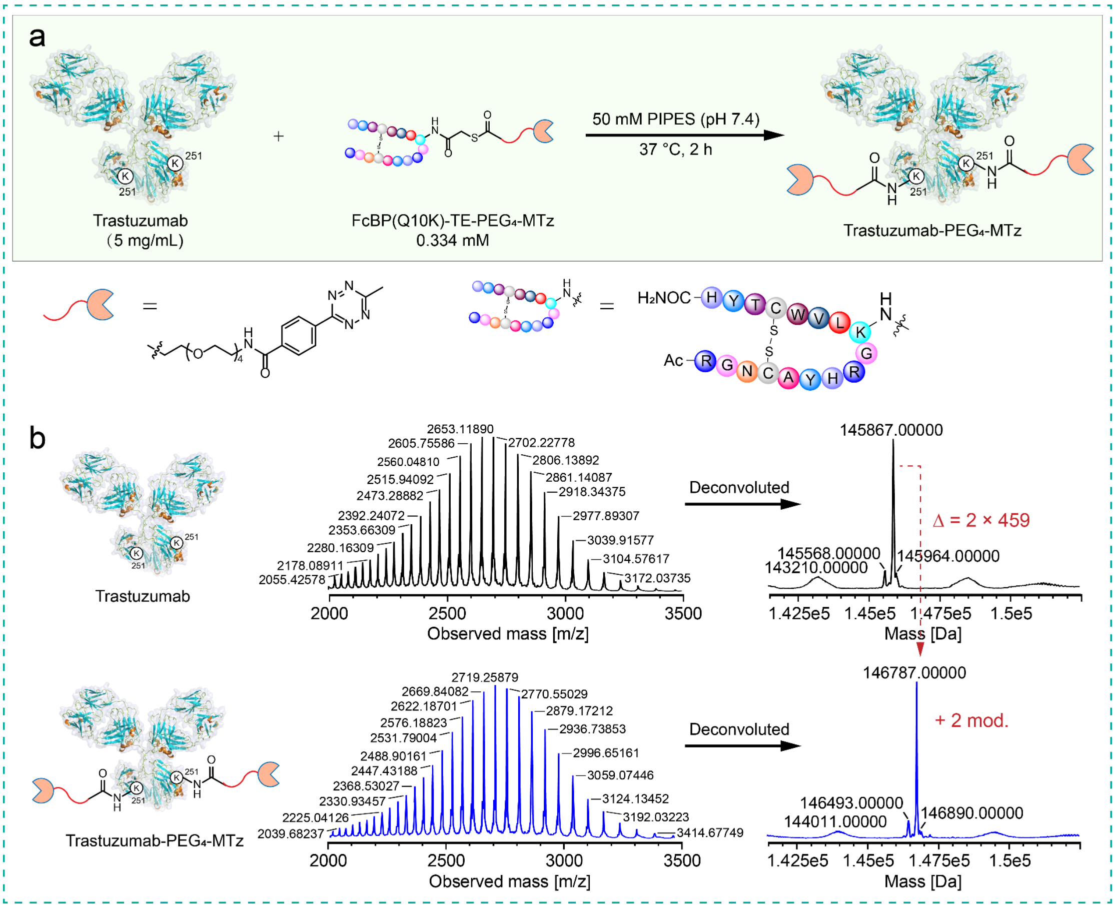

Supplementary Figure 24. Synthesis of MTz-tagged Trastuzumab by FcBP(Q10K)-TE-PEG4-MTz. **a.** Scheme of one-step synthesis of MTz-tagged Trastuzumab. **b.** LC-MS profiles of Trastuzumab (upper) and MTz-tagged Trastuzumab (lower).

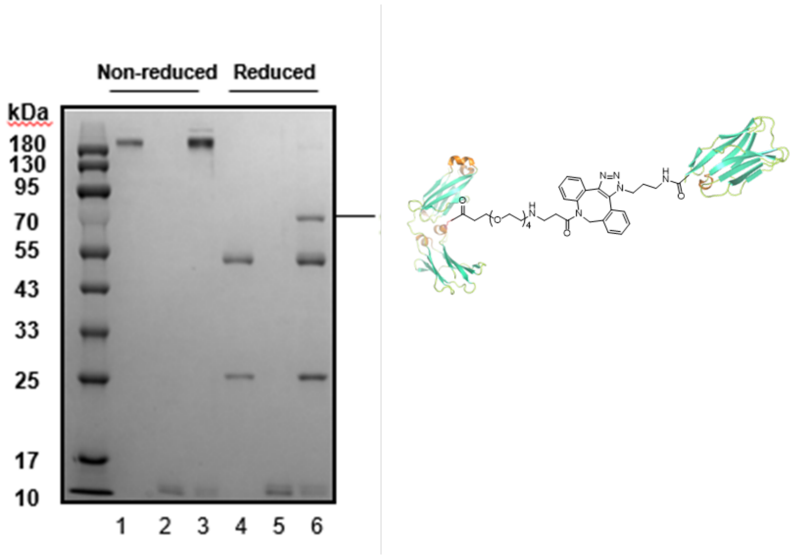

Supplementary Figure 25. SDS-PAGE analysis of the assembly of Tras-Her2-Nb biparatopic antibodies. Lane M, molecular weight marker; Lane 1, Tras-PEG4-DBCO; Lane 2, Her2 Nb-Az, Lane 3, incubation of Tras-PEG4-DBCO and Her2 Nb-Az and lanes 4-6, reduced gels of lanes 1-3.

Supplementary Table 1. Observed and calculated masses of CPD-tagged proteins and their conjugates.

Protein	Calculated average mass (Da)	Observed mass (Da)
Endo-F3(D165A)-CPD	55616.5	55616.0
Endo-F3(D165A)-COOH	31888.8	31888.0
Endo-F3(D165A)-azide	31970.9	31971.0
Endo-F3(D165A)-OH (Tertiary alcohol)	31959.9	31960
Endo-F3(D165A)-alkynyl	31925.8	31926.0
Endo-F3(D165A)-Gly	31945.8	31946.0
Endo-F3(D165A)-Gly-Gly	32002.9	32003.0
Endo-F3(D165A)-SO_3H	32009.9	32010.0
Endo-F3(D165A)-OH (Primary alcohol)	31959.9	31960
Endo-F3(D165A)-SH	31947.9	31948.0
Endo-F3(D165A)-NH_2	31930.9	31931.0
Endo-F3(D165A)-$NHNH_2$	31902.8	31903.0
Endo-F3(D165A)-Biotin	32157.1	32157.0
Endo-F3(D165A)-PEG_3-alkynyl	32058.0	32058.0
Endo-F3(D165A)-Gly-Gly-Gly	32059.9	32060.0
Endo-F3(D165A)-PEG_2-azide	32045.0	32045.0
Her2 Nb-CPD	36997.5	36998.0
Her2 Nb-COOH	13269.9	13270.0
Her2 Nb-azide	13352.0	13351.0
Her2 Nb-FITC	14017.7	14017.0
Her2 Nb-Lys(PEG_{24})-VC-PAB-MMAE	15923.1	15923.0
Her2 Nb-$NHNH_2$	13283.9	13284.0
Her2 Nb-GlcNAc	13487.1	13487.0
Her2 Nb-Cys	13373.0	13373.0
Her2 Nb-Cys(MPAA)-Flag	14534.2	14534.0
GFP-CPD	49819.4	49815.0
GFP-COOH	26091.6	26087.0
GFP-azide	26173.1	26170.0
GFP-alkynyl	26128.7	26124.0
GST-CPD	49624.6	49624.0
GST-COOH	25896.9	25896.0
GST-azide	25979.0	25978.0
Endo-A-CPD	93822.9	93752.0
Endo-A-COOH	70095.1	70023.0
Endo-A-Azide	70177.2	70106.0
Endo-S2-CPD	109850.0	109854.0

Endo-S2-COOH	86122.2	86123.0
Endo-S2-Azide	86204.3	86207.0
Flag-Her2 Nb-CPD	37996.5	37994.0
Flag-Her2 Nb-COOH	14268.7	14265.0
Flag-Her2 Nb-PEG_4-TCO	14638.9	14636.0
CPD	23745.8	23745.0

2. General Information and Procedures

High Performance Liquid Chromatography (HPLC).

Method A: Analytical RP-HPLC was performed on a Thermo ultimate 3000 instrument with a C18 column (Agilent, 4 μm, 4.6 x 150 mm) at 40 °C. The column was eluted with a linear gradient of 2-90% acetonitrile containing 0.25% TFA in 30 min at a flow rate of 1 mL/min.

Method B: Semi-preparative HPLC was performed on a Beijing ChuangXinTongHeng LC3000 (preparative) instrument with a preparative C-18 column (Waters, 5 μm, 19 x 250 mm). The column was eluted with a suitable gradient of aqueous acetonitrile containing 0.25% TFA at a flow rate of 8 mL/min.

Liquid Chromatography and Electron Spray Ionization Mass Spectrometry (LC-ESI-MS).

Electrospray ionization mass spectrometry (ESI−MS) was performed on a Waters Xevo G2-XS Q-TOF. Method: mobile phase A = 0.1% formic acid water; mobile phase B = 0.1% formic acid acetonitrile; gradient 0-1.3 min, 10%-70% phase B; flow rate = 0.3 mL/ min. Detecting absorbance is 214 nm. The small molecules were analyzed using a Waters C18 column (ACQUITY UPLC BEH C18, 1.7 μm, 2.1 x 50 mm).

3. Experimental section

3.1 Optimization of the Reaction Conditions.

Labeling reactions were conducted in PBS, PB, HEPES, Tris, MOPS, Tricine and NaOAc buffer (50 mM) containing Endo-F3(D165A)-CPD at 100 μM, 3-adizopropylamine at 2-50 mM, InsP$_6$ at 0.2-500 μM, and DMAP at 0-10 mM (final pH=8.0) at 4°C or 25°C. The reaction time course was done by quenching reactions via TFA addition to 0.1% (v/v), and monitored by ESI-TOF-MS.

3.2 Validation test of CPD-mediated hydrolyze.

Test 1 were conducted in PBS containing Endo-F3(D165A)-azido (Endo-F3(D165A)-Az) at 40 μM, with/without 80 μM InsP$_6$, 80 μM CPD at room temperature for 12 h. The progress of the reaction was monitored by ESI-TOF-MS.

Table S2 Condition of Validation test 1

Entry	Endo-F3(D165A)-Az	InsP$_6$	CPD
1	40 μM	80 μM	80 μM
2	40 μM	-	-
3	40 μM	-	80 μM
4	40 μM	80 μM	-

Test 2 were conducted in PBS containing Endo-F3(D165A)-CPD-S-R (The thiol on CPD was blocked by R, R = SMCC-PEG$_4$-FcBP) at 22 μM, with/without 100 μM InsP$_6$, 22 μM CPD, or 22 μM CPD-tagged Endo-F3(D165A) (Endo-F3(D165A)-CPD-SH) at room temperature for 5 h. The progress of the reaction was monitored by ESI-TOF-MS.

Table S3 Condition of Validation test 2

Entry	Endo-F3(D165A)-CPD-S-R	InsP$_6$	CPD	Endo-F3(D165A)-CPD-SH
1	22 μM	100 μM	-	-
2	22 μM	100 μM	22 μM	-
3	22 μM	100 μM	-	22 μM

3.3 Synthesis

3.3.1 Synthesis of Biotin-NH$_2$ (S1)

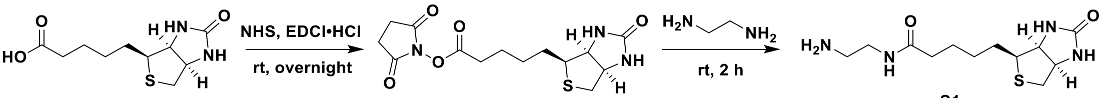

S1

To a solution of Biotin (20 mg, 81.97 μmol) in DMF (200 μL) was added N-hydroxysuccinimide (11.3 mg, 98.36 μmol) and EDCI▪HCl (18.8 mg, 98.36 μmol). The reaction mixture was stirred at room temperature for overnight. Then ethylenediamine (27 μL, 0.41 mM) was added and the mixture was stirred at room temperature for 2 h. The mixture was purified by semi-preparative HPLC to get **S1** as a white powder (85%). [M+H]$^+$ calculated for $C_{12}H_{22}N_4O_2S$: 287.1542; found m/z=287.1545. ^{1}H NMR (500 MHz, D_2O) δ 4.64 – 4.60 (m, 1H), 4.43 (dd, J = 8.0, 4.5 Hz, 1H), 3.50 (t, J = 6.1 Hz, 2H), 3.35 (dt, J = 9.9, 5.1 Hz, 1H), 3.15 (t, J = 6.1 Hz, 2H), 3.00 (dd, J = 13.1, 5.0 Hz, 1H), 2.79 (d, J = 13.0

Hz, 1H), 2.31 (t, J = 7.4 Hz, 2H), 1.78 – 1.54 (m, 4H), 1.43 (dddd, J = 12.9, 11.3, 7.7, 4.9 Hz, 2H). ^{13}C NMR (126 MHz, D_2O) δ 177.88, 165.37, 62.06, 60.25, 55.31, 39.63, 39.11, 36.70, 35.28, 27.89, 27.62, 24.80.

Synthesis of peptide H-CDYKDDDK-OH

Peptide H-CDYKDDDK-OH was synthesized by standard solid phase peptide synthesis (SPPS) on 2-Chlorotrityl Chloride Resin (loading: 1.311 mmol/g). Amino acid couplings were done using 4 equivalents of amino acid with 4 equivalents of HATU and 8 equivalents of DIPEA in DMF. Fmoc removal was accomplished by incubating the resin with a 20% solution of piperidine in DMF for 15 min.

3.3.2 Synthesis of DBCO-FITC (S2)

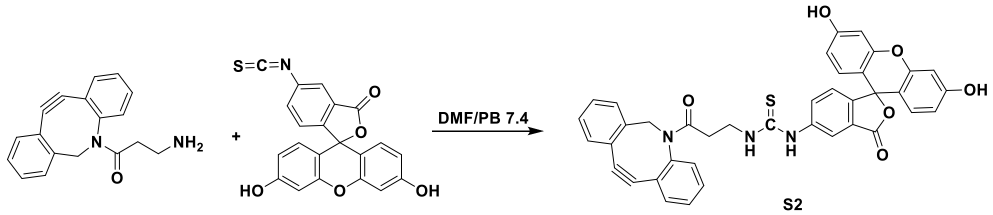

To a stirred solution of DBCO-amine (10 mg, 36.23 μM) in DMF/0.2 M PB 7.4=1:1 was added FITC (14 mg, 36.23 μM), the reaction mixture was stirred at room temperature and in dark for 2 h, The mixture was purified by semi-preparative HPLC to get **S2** as a yellow powder (77%). $[M+H]^+$ calculated for $C_{39}H_{27}N_3O_6S$: 666.1699; found m/z=666.1723. ^{1}H NMR (500 MHz, DMSO-*d*6) δ 9.93 (s, 1H), 8.17 (s, 1H), 7.96 (s, 1H), 7.68 – 7.61 (m, 3H), 7.54 – 7.45 (m, 3H), 7.38 (td, J = 7.4, 1.6 Hz, 1H), 7.35 – 7.27 (m, 2H), 7.11 (d, J = 8.3 Hz, 1H), 6.67 (d, J = 2.2 Hz, 2H), 6.62 – 6.53 (m, 5H), 5.07 (d, J = 14.1 Hz, 1H), 3.67 (d, J = 14.0 Hz, 1H), 3.59 – 3.46 (m, 2H), 2.70 (dt, J = 16.1, 7.0 Hz, 1H), 1.98 (dt, J = 16.3, 6.5 Hz, 1H). ^{13}C NMR (126 MHz, DMSO-*d*6) δ 179.61, 170.04, 168.00, 159.00, 151.38, 150.72, 147.89, 146.64, 140.69, 131.88, 129.14, 128.54, 127.83, 127.59, 127.25, 126.37, 125.96, 124.76, 123.55, 123.52, 121.98, 120.99, 115.91, 113.76, 112.09, 109.22, 107.55, 101.73, 54.49, 33.02.

3.3.3 Synthesis of BCN-Lys(PEG24)-VC-PAB-MMAE (S6)

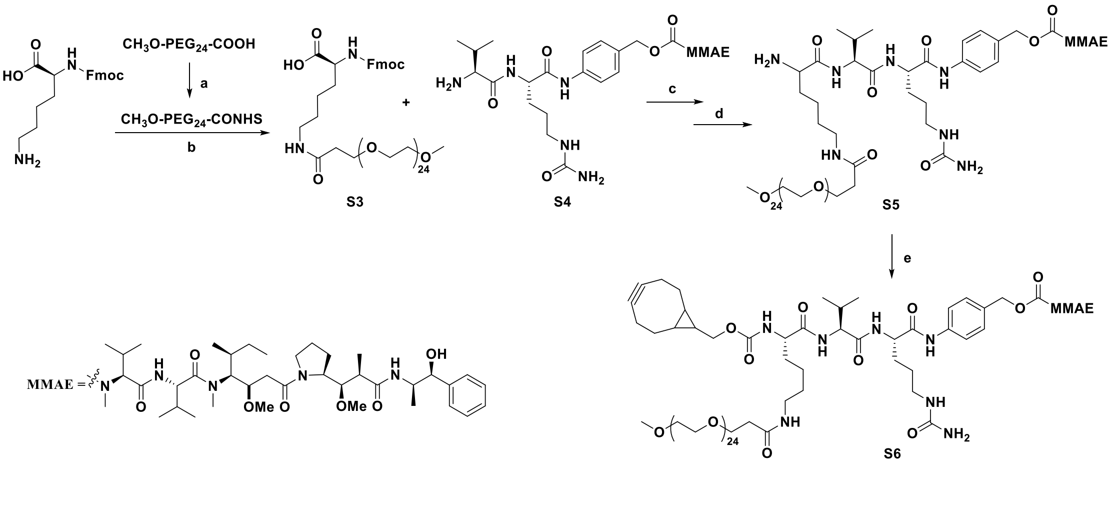

Reagents and conditions: (a) CH_3O-PEG_{24}-COOH (1 eq), NHS (1.2 eq), EDC (1.2 eq), ACN, room temperature, overnight. (b) CH_3O-PEG_{24}-CONHS (1 eq), Fmoc-Lys(NH_2)-OH (1 eq), ACN, room temperature, 2 h. (c) **S4** (1 eq), HATU (2 eq), DIPEA (3 eq), **S3** (1 eq), DMF, room temperature, 2 h. (d) 20 % piperidine, DMF, room temperature, 15 min. (e) **S5** (1 eq), BCN-O-PNP (1.2 eq), Et_3N (3 eq), DMF, room temperature, 2h.

Synthesis of **S3**. To a stirred solution of CH_3O-PEG_{24}-COOH (58 mg, 0.05 mmol) in ACN was added N-hydroxysuccinimide (6.8 mg, 0.06 mmol) and EDC (7.9 μL, 0.06 mmol). The reaction mixture was stirred at room temperature for overnight. Then a solution of Fmoc-Lys(NH_2)-OH (18.4 mg, 0.05 mmol) dissolved in ACN was added and the mixture was stirred at room temperature for 2 h. The mixture was purified by semi-preparative HPLC to get a white powder (78%). $[M+H]^+$ calculated for $C_{73}H_{126}N_2O_{30}$: $[M+H]^+$ 756.4275 , found m/z=756.4218.

Synthesis of **S5**. To a stirred solution of **S4** (13.5 mg, 8.9 μmol) in DMF (400 μL), HATU (6.8 mg, 17.8 μmol), DIPEA (4.6 μL, 27.6 μmol) and **S3** (10 mg, 8.9 μmol) were added and the reaction mixture was stirred at room temperature for 2 h and monitored by LC-MS. Then the piperidine (100 μL) was added and the mixture was stirred at room temperature for 15 min. Then the solution was purified by semi-preparative HPLC to get **S5** as a white powder (86%). $[M+H]^+$ calculated for $C_{116}H_{208}N_{12}O_{39}$: $[M+H]^+$ 2394.4741, $[M+3H]^{3+}$ 798.8299, found m/z=798.8312.

Synthesis of **S6**. To a stirred solution of **S5** (10 mg, 4.2 μmol) in DMF was added BCN-O-PNP (1.63 mg, 5 μmol) and Et_3N (1.7 μL, 12.6 μmol). The mixture was stirred at room temperature for 2 h and monitored by LC-MS. Then the solution was purified by semi-preparative HPLC to get **S6** as a white powder (76%). $[M+H]^+$ calculated for $C_{116}H_{208}N_{12}O_{39}$: $[M+H]^+$ 2570.5577, $[M+3H]^{3+}$ 857.5245, found m/z=857.5310.

3.3.4 Synthesis of FcBP(Q10K)-TE-PEG4-DBCO (S12)

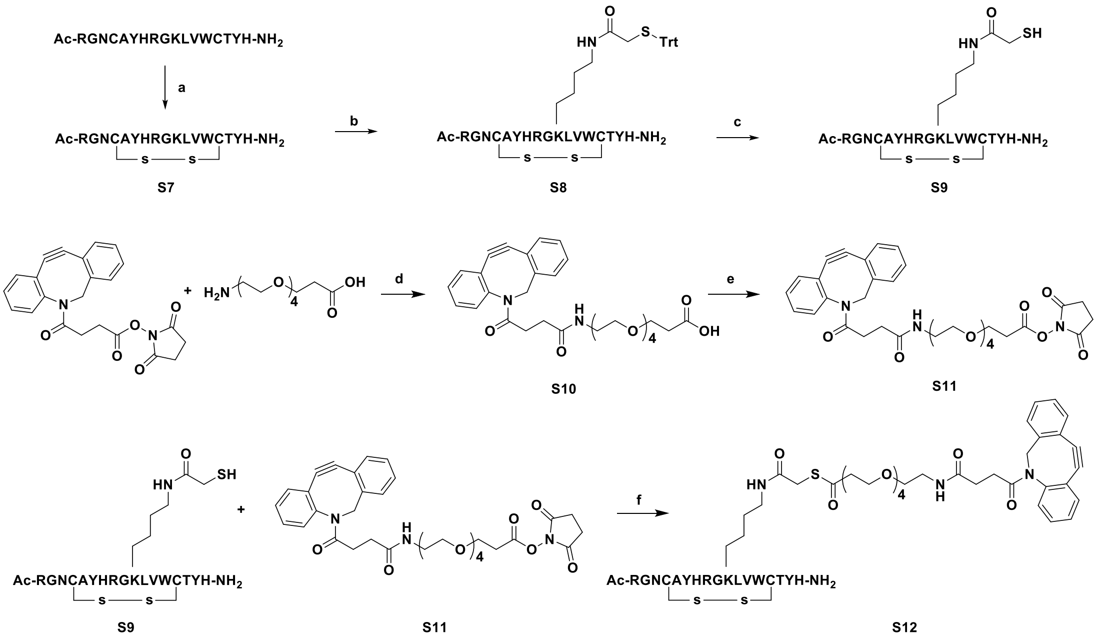

Reagents and conditions: (a) H_2O_2 (2 eq), NH_3-H_2O (20 eq), DMSO, room temperature, overnight. (b) 2-(triphenylmethylthio)ethanoic acid (4 eq), HATU (2 eq), DIPEA (3 eq), **S7** (1 eq), DMF, room temperature, 1 h. (c) DCM/TFA/TIPS=50/45/5, room temperature, 1 h. (d) Et_3N (6 eq), DMF, room temperature, 2 h. (e) NHS (1.2 eq), EDCI•HCl (1.2 eq), DMF, room temperature, overnight. (f) Et_3N (3 eq), DMF, room temperature, 2 h.

Synthesis of cyclic peptide **S7**

To a stirred solution of peptide **Ac-RGNCAYHRGKLVWCTYH-NH$_2$** (5 mM) in DMSO was added H_2O_2 (10 mM) and NH_3-H_2O (100 mM) respectively, the mixture was stirred at room temperature for overnight. The mixture was purified by semi-preparative HPLC to get **S7** as a white powder (90%). $[M+H]^+$ calculated for $C_{93}H_{136}N_{31}O_{22}S_2$: $[M+2H]^{2+}$ 1051.9998, $[M+3H]^{3+}$ 701.6691, found m/z=1052.0009, 701.6714.

Synthesis of FcBP (Q10K)-SH **S9**

To a stirred solution of 2-(triphenylmethylthio)ethanoic acid (6.4 mg, 19.04 μmol) in DMF, HATU (3.6 mg, 9.52 μmol), DIPEA (2.45 μL, 14.28 μmol) and **S7** (10.0 mg, 4.76 μmol) were added and the reaction mixture was stirred at room temperature for 1 h. Then the solution was purified by semi-preparative HPLC to get a white powder (95%). $[M+H]^+$ calculated for $C_{114}H_{151}N_{31}O_{23}S_3$: $[M+2H]^{2+}$ 1210.0459, $[M+3H]^{3+}$ 807.0332, $[M+4H]^{4+}$ 605.5269, found m/z= 1210.0478, 807.0341, 605.5315. To a stirred solution of the intermediate in dichloromethane (250 μL) and cooled to 0 °C (ice bath). The colorless solution was treated with 225 μL of trifluoroacetic acid, and next, triisopropylsilane (25 μL) was immediately added to the reaction mixture and stirred for 1 h. After which time, the mixture was concentrated to afford crude **S9**. $[M+H]^+$ calculated for $C_{95}H_{137}N_{31}O_{23}S_3$: $[M+2H]^{2+}$ 1088.9911, $[M+3H]^{3+}$ 726.3300, $[M+4H]^{4+}$ 544.9995, found m/z=1089.0125, 726.3401, 545.0055.

Synthesis of DBCO-PEG_4-COOH **S10**

To a stirred solution of Dibenzocyclooctyne-N-hydroxysuccinimidyl ester (DBCO-CONHS, 20.0 mg, 49.75 μmol) in DMF, α-amine-ω-propionic acid tetraethylene glycol (13.2 mg, 49.75 μmol) and Et_3N (41.4 μL, 298.5 μmol) were added and the reaction mixture was stirred at room temperature for 2 h. Then the solution was purified by semi-preparative HPLC to get **S10** as a yellow solid (90%). $[M+H]^+$ calculated for $C_{30}H_{36}N_2O_8$: $[M+H]^+$ 553.2550, found m/z=553.2070.

Synthesis of DBCO-PEG_4-NHS ester **S11**

To a solution of **S10** (24.7 mg, 44.78 μmol) in DMF (200 μL) was added N-hydroxysuccinimide (6.2 mg, 53.73 μmol) and EDCI▪HCl (10.3 mg, 53.73 μmol). The reaction mixture was stirred at room temperature for overnight. Then the solution was purified by semi-preparative HPLC to get **S11** as a yellow solid (86%). $[M+H]^+$ calculated for $C_{34}H_{39}N_3O_{10}$: $[M+H]^+$ 650.2714, found m/z=650.2745. ^1H NMR (500 MHz, DMSO-d_6) δ 7.89 (t, *J* = 5.7 Hz, 1H), 7.80 (d, *J* = 7.5 Hz, 1H), 7.60 (dd, *J* = 7.7, 4.4 Hz, 2H), 7.47 – 7.42 (m, 2H), 7.37 (td, *J* = 7.5, 1.2 Hz, 1H), 7.14 (ddd, *J* = 8.2, 7.0, 1.1 Hz, 1H), 7.03 (ddd, *J* = 7.9, 7.0, 1.0 Hz, 1H), 6.67 (s, 1H), 3.71 (d, *J* = 5.9 Hz, 2H), 3.50 (d, *J* = 2.9 Hz, 12H), 3.38 (d, *J* = 5.9 Hz, 2H), 3.18 (d, *J* = 5.8 Hz, 2H), 2.91 (d, *J* = 5.9 Hz, 2H), 2.81 (s, 6H), 2.42 – 2.38 (m, 2H), 2.31 (t, *J* = 6.7 Hz, 2H). ^{13}C NMR (126 MHz, DMSO-d_6) δ 174.31, 171.49, 170.60, 167.83, 143.83, 142.63, 133.99, 132.63, 128.52, 127.73, 124.53, 121.71, 121.61, 121.16, 119.81, 110.35, 91.41, 70.25, 70.24, 70.17, 70.13, 70.05, 69.58, 65.69, 48.84, 39.04, 32.06, 30.37, 29.58, 25.91.

Synthesis of FcBP (Q10K)-TE-PEG_4-DBCO **S12**

To a stirred solution of FcBP (Q10K)-SH **S9** (12.5 mM) in DMF was added DBCO-PEG_4-NHS ester **S11** (12.5 mM) and Et_3N (37.5 mM) respectively, the mixture was stirred at room temperature for 2 h. Then the solution was purified by semi-preparative HPLC to get **S12** as a white powder (80%). $[M+H]^+$ calculated for $C_{125}H_{171}N_{33}O_{30}S_3$: $[M+2H]^{2+}$ 1356.1094, $[M+3H]^{3+}$ 904.4089, $[M+4H]^{4+}$ 678.5586, found m/z=1356.1107, 904.4077, 678.5548.

3.3.5 Synthesis of FcBP(Q10K)-TE-PEG$_4$-MTz (S15)

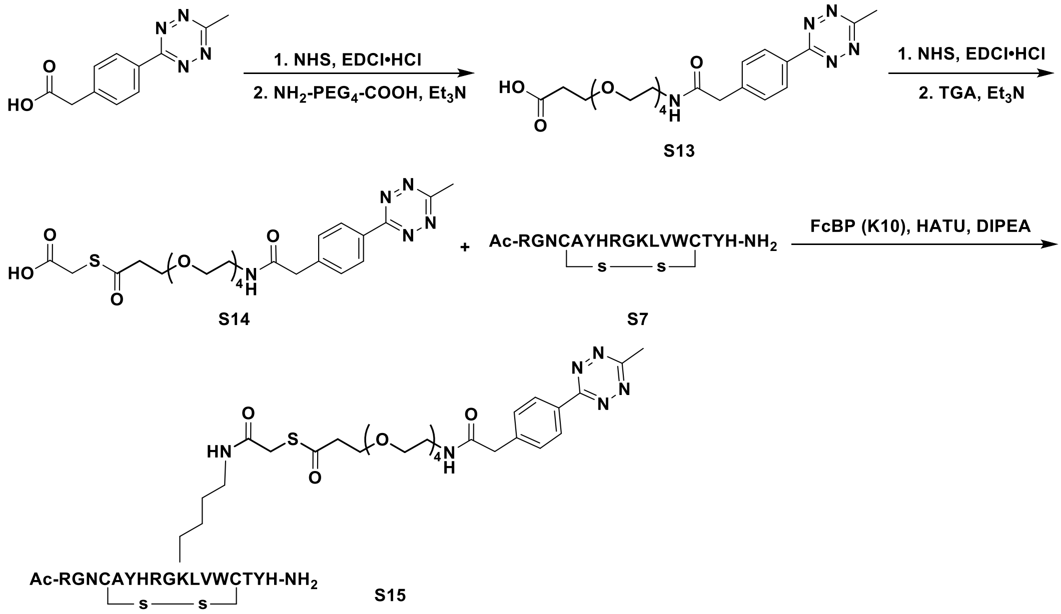

Synthesis of MTz-PEG$_4$-COOH **S13**

To a stirred solution of methyltetrazine-acid (10.0 mg, 43.5 μmol) in DMF (600 μL), N-hydroxysuccinimide (12.6 mg, 110 μmol) and EDCI•HCl (21 mg, 110 μmol) were added and the reaction mixture was stirred at room temperature for overnight. Then a solution of α-amine-ω-propionic acid tetraethylene glycol (11.6 mg, 43.5 μmol) and Et_3N (18.3 μL, 132 μmol) dissolved in DMF was added and the mixture was stirred at room temperature for 3 h. The mixture was purified by semi-preparative HPLC to get **S13** as a purple powder (86%). $[M+H]^+$ calculated for $C_{22}H_{31}N_5O_7$: $[M+H]^+$ 478.2302 , found m/z=478.2318.

Synthesis of MTz-PEG$_4$-thioester **S14**

To a stirred solution of MTz-PEG$_4$-COOH **S13** (9.5 mg, 20 μmol) in DMF (300 μL), N-hydroxysuccinimide (2.8 mg, 24 μmol) and EDCI•HCl (4.6 mg, 24 μmol) were added and the reaction mixture was stirred at room temperature for overnight. Then the mercaptoacetic acid (6.9 μL, 100 μmol) and Et_3N (8.3 μL, 60 μmol) were added, the mixture was stirred at room temperature for 30 min. The mixture was purified by semi-preparative HPLC to get **S14** as a purple powder (53%). $[M+H]^+$ calculated for $C_{24}H_{33}N_5O_8S$: $[M+H]^+$ 552.2128 , found m/z=552.2157.

Synthesis of FcBP (Q10K)-TE-PEG$_4$-MTz **S15**

To a stirred solution of MTz-PEG$_4$-thioester **S14** (2.5 mg, 4.6 μmol) in DMF (100 μL), HATU (3.5 mg, 9.2 μmol), DIPEA (2.37 μL, 13.8 μmol) and **S7** (9.7 mg, 4.6 μmol) were added and the reaction mixture was stirred at room temperature for 2 h and monitored by LC-MS. The mixture was purified by semi-preparative HPLC to get **S15** as a purple powder (63%). $[M+H]^+$ calculated for $C_{117}H_{166}N_{36}O_{29}S_3$: $[M+H]^+$ 2636.1862, $[M+2H]^{2+}$ 1318.5970, $[M+3H]^{3+}$ 879.4006, $[M+4H]^{4+}$ 659.8024, found m/z=1318.5944, 879.4110, 659.8125..

3.3.6 Synthesis of DBCO-tagged Trastuzumab

A solution of Trastuzumab (5 mg/mL), FcBP(Q10K)-TE-PEG$_4$-DBCO (0.2 mM, 6 eq) and

3-maleimideopropionic acid (0.2 mM, 6 eq) in 50 mM PIPES buffer (pH 7.4) containing 20% DMF was incubated at 37 °C for 2 h. The progress of the reaction was monitored by ESI-TOF-MS. The reaction mixture was purified by affinity chromatography via protein A resin and concentrated by centrifugal filtration (30 kDa, Millipore). The buffer was exchanged for PBS buffer to give the DBCO-tagged antibody.

3.3.7 Synthesis of MTz-tagged Trastuzumab

A solution of Trastuzumab (5 mg/mL), FcBP(Q10K)-TE-PEG_4-MTz (0.334 mM, 10 eq) and 3-maleimideopropionic acid (0.2 mM, 6 eq) in 50 mM PIPES buffer (pH 7.4) containing 20% DMF was incubated at 37 °C for 2 h. The progress of the reaction was monitored by ESI-TOF-MS. The reaction mixture was purified by affinity chromatography via protein A resin and concentrated by centrifugal filtration (30 kDa, Millipore). The buffer was exchanged for PBS buffer to give the MTz-tagged antibody.

3.3.8 Synthesis of Flag-Her2 nanobody-PEG_4-TCO

General labeling reactions were conducted in NaOAc buffer (50 mM, pH 7.4) containing Flag-Her2 nanobody-CPD at 100 μM, TCO-PEG_4-NH_2 at 5 mM, $InsP_6$ at 5 μM, and DMAP at 5 mM (final pH=8.0) at 4°C for 5 h. The progress of the reaction was monitored by ESI-TOF-MS. The reaction mixture was purified by affinity chromatography via the anti-DYKDDDDK G1 Affinity Resin and concentrated by centrifugal filtration (3 kDa, Millipore). The buffer was exchanged for PBS buffer to give the Flag-Her2 nanobody-PEG_4-TCO.

4. Protein Sequences

4.1 Her2 Nb-CPD

Protein sequence of CPD-tagged Her2 nanobody:

MQVQLQESGGGSVQAGGSLKLTCAASGYIFNSCGMGWYRQSPGRERELVSRISGDGD
TWHKESVKGRFTISQDNVKKTLYLQMNSLKPEDTAVYFCAVCYNLETYWGQGTQVTVS
SGGVDALADGKILHNQNVNSWGPITVTPTTDGGETRFDGQIIVQMENDPVVAKAAANLA
GKHAESSVVVQLDSDGNYRVVYGDPSKLDGKLRWQLVGHGRDHSETNNTRLSGYSAD
ELAVKLAKFQQSFNQAENINNKPDHISIVGCSLVSDDKQKGFGHQFINAMDANGLRVDV
SVRSSELAVDEAGRKHTKDANGDWVQKAENNKVSLSWDAQGLEHHHHHH

DNA sequence of CPD-tagged Her2 nanobody:

ATGCAAGTTCAGCTGCAGGAAAGCGGTGGTGGTAGCGTTCAGGCCGGTGGTAGCCT
GAAACTGACCTGCGCCGCCAGCGGTTATATTTTTAATAGCTGCGGTATGGGTTGGTAT
CGTCAGAGTCCGGGTCGTGAACGTGAACTGGTTAGCCGTATTAGCGGTGATGGTGA
TACCTGGCATAAAGAAAGCGTGAAAGGTCGTTTTACCATTAGCCAGGATAATGTGAAG
AAAACCCTGTATCTGCAGATGAATAGCCTGAAACCGGAAGATACCGCCGTGTATTTTT
GCGCCGTTTGCTATAATCTGGAAACCTATTGGGGTCAGGGTACCCAGGTGACCGTGA
GCAGCGGTGGTGTCGACGCATTAGCGGATGGAAAAATACTCCATAATCAAAATGTTAA
TAGCTGGGGCCCGATTACGGTTACACCAACGACAGATGGTGGTGAAACCCGCTTCG
ACGGTCAAATCATCGTTCAAATGGAAAACGACCCGGTAGTAGCAAAAGCGGCAGCCA
ATTTAGCAGGTAAACATGCTGAAAGCAGTGTGGTGGTGCAGCTCGATTCAGACGGCA
ACTATCGCGTGGTGTATGGCGATCCGTCAAAACTGGATGGAAAGCTACGTTGGCAGT
TGGTGGGGCATGGTCGCGACCACTCAGAAACTAACAATACTCGCTTAAGTGGTTACA
GTGCCGATGAGTTGGCCGTGAAATTGGCCAAGTTCCAACAGTCGTTTAATCAAGCCG
AAAACATCAACAACAAACCGGATCACATCAGTATTGTTGGTTGTTCTTTGGTGAGTGA
CGACAAGCAAAAAGGCTTTGGTCATCAGTTTATTAACGCGATGGATGCGAATGGTCTT
CGTGTCGATGTCTCTGTTCGTAGTTCTGAACTGGCCGTAGACGAGGCGGGACGTAA
GCATACCAAGGACGCGAATGGCGATTGGGTTCAAAAGGCAGAAAACAACAAAGTTTC
GCTAAGCTGGGACGCGCAAGGTCTCGAGCACCACCACCACCACCAC

4.2 GST-CPD

Protein sequence of CPD-tagged GST:

MSPILGYWKIKGLVQPTRLLLEYLEEKYEEHLYERDEGDKWRNKKFELGLEFPNLPYYID
GDVKLTQSMAIIRYIADKHNMLGGCPKERAEISMLEGAVLDIRYGVSRIAYSKDFETLKVD
FLSKLPEMLKMFEDRLCHKTYLNGDHVTHPDFMLYDALDVVLYMDPMCLDAFPKLVCFK
KRIEAIPQIDKYLKSSKYIAWPLQGWQATFGGGDHPPKVDALADGKILHNQNVNSWGPIT
VTPTTDGGETRFDGQIIVQMENDPVVAKAAANLAGKHAESSVVVQLDSDGNYRVVYGD
PSKLDGKLRWQLVGHGRDHSETNNTRLSGYSADELAVKLAKFQQSFNQAENINNKPDH
ISIVGCSLVSDDKQKGFGHQFINAMDANGLRVDVSVRSSELAVDEAGRKHTKDANGDW
VQKAENNKVSLSWDAQGLEHHHHHH

DNA sequence of CPD-tagged GST:

ATGTCCCCTATACTAGGTTATTGGAAAATTAAGGGCCTTGTGCAACCCACTCGACTTC

TTTTGGAATATCTTGAAGAAAAATATGAAGAGCATTTGTATGAGCGCGATGAAGGTGAT
AAATGGCGAAACAAAAAGTTTGAATTGGGTTTGGAGTTTCCCAATCTTCCTTATTATAT
TGATGGTGATGTTAAATTAACACAGTCTATGGCCATCATACGTTATATAGCTGACAAGC
ACAACATGTTGGGTGGTTGTCCAAAAGAGCGTGCAGAGATTTCAATGCTTGAAGGAG
CGGTTTTGGATATTAGATACGGTGTTTCGAGAATTGCATATAGTAAAGACTTTGAAACT
CTCAAAGTTGATTTTCTTAGCAAGCTACCTGAAATGCTGAAAATGTTCGAAGATCGTT
TATGTCATAAAACATATTTAAATGGTGATCATGTAACCCATCCTGACTTCATGTTGTATG
ACGCTCTTGATGTTGTTTTATACATGGACCCAATGTGCCTGGATGCGTTCCCAAAATT
AGTTTGTTTTAAAAAACGTATTGAAGCTATCCCACAAATTGATAAGTACTTGAAATCCA
GCAAGTATATAGCATGGCCTTTGCAGGGCTGGCAAGCCACGTTTGGTGGTGGCGAC
CATCCTCCAAAAGTCGACGCATTAGCGGATGGAAAAATACTCCATAATCAAAATGTTAA
TAGCTGGGGCCCGATTACGGTTACACCAACGACAGATGGTGGTGAAACCCGCTTCG
ACGGTCAAATCATCGTTCAAATGGAAAACGACCCGGTAGTAGCAAAAGCGGCAGCCA
ATTTAGCAGGTAAACATGCTGAAAGCAGTGTGGTGGTGCAGCTCGATTCAGACGGCA
ACTATCGCGTGGTGTATGGCGATCCGTCAAAACTGGATGGAAAGCTACGTTGGCAGT
TGGTGGGGCATGGTCGCGACCACTCAGAAACTAACAATACTCGCTTAAGTGGTTACA
GTGCCGATGAGTTGGCCGTGAAATTGGCCAAGTTCCAACAGTCGTTTAATCAAGCCG
AAAACATCAACAACAAACCGGATCACATCAGTATTGTTGGTTGTTCTTTGGTGAGTGA
CGACAAGCAAAAAGGCTTTGGTCATCAGTTTATTAACGCGATGGATGCGAATGGTCTT
CGTGTCGATGTCTCTGTTCGTAGTTCTGAACTGGCCGTAGACGAGGCGGGACGTAA
GCATACCAAGGACGCGAATGGCGATTGGGTTCAAAAGGCAGAAAACAACAAAGTTTC
GCTAAGCTGGGACGCGCAAGGTCTCGAGCACCACCACCACCACCAC

4.3 GFP-CPD

Protein sequence of CPD-tagged GFP:
MESDESGLPAMEIECRITGTLNGVEFELVGGGEGTPEQGRMTNKMKSTKGALTFSPYLL
SHVMGYGFYHFGTYPSGYENPFLHAINNGGYTNTRIEKYEDGGVLHVSFSYRYEAGRVI
GDFKVMGTGFPEDSVIFTDKIIRSNATVEHLHPMGDNDLDGSFTRTFSLRDGGYYSSVV
DSHMHFKSAIHPSILQNGGPMFAFRRVEEDHSNTELGIVEYQHAFKTPDADAGEEVDAL
ADGKILHNQNVNSWGPITVTPTTDGGETRFDGQIIVQMENDPVVAKAAANLAGKHAESS
VVVQLDSDGNYRVVYGDPSKLDGKLRWQLVGHGRDHSETNNTRLSGYSADELAVKLA
KFQQSFNQAENINNKPDHISIVGCSLVSDDKQKGFGHQFINAMDANGLRVDVSVRSSEL
AVDEAGRKHTKDANGDWVQKAENNKVSLSWDAQGLEHHHHHH

DNA sequence of CPD-tagged GFP:
ATGGAGAGCGACGAGAGCGGCCTGCCCGCCATGGAGATCGAGTGCCGCATCACCG
GCACCCTGAACGGCGTGGAGTTCGAGCTGGTGGGCGGCGGAGAGGGCACCCCCG
AGCAGGGCCGCATGACCAACAAGATGAAGAGCACCAAAGGCGCCCTGACCTTCAG
CCCCTACCTGCTGAGCCACGTGATGGGCTACGGCTTCTACCACTTCGGCACCTACC
CCAGCGGCTACGAGAACCCCTTCCTGCACGCCATCAACAACGGCGGCTACACCAAC
ACCCGCATCGAGAAGTACGAGGACGGCGGCGTGCTGCACGTGAGCTTCAGCTACC
GCTACGAGGCCGGCCGCGTGATCGGCGACTTCAAGGTGATGGGCACCGGCTTCCC
CGAGGACAGCGTGATCTTCACCGACAAGATCATCCGCAGCAACGCCACCGTGGAGC
ACCTGCACCCCATGGGCGATAACGATCTGGATGGCAGCTTCACCCGCACCTTCAGC

CTGCGCGACGGCGGCTACTACAGCTCCGTGGTGGACAGCCACATGCACTTCAAGAG
CGCCATCCACCCCAGCATCCTGCAGAACGGGGGCCCCATGTTCGCCTTCCGCCGC
GTGGAGGAGGATCACAGCAACACCGAGCTGGGCATCGTGGAGTACCAGCACGCCT
TCAAGACCCCGGATGCAGATGCCGGTGAAGAAGTCGACGCATTAGCGGATGGAAAA
ATACTCCATAATCAAAATGTTAATAGCTGGGGCCCGATTACGGTTACACCAACGACAG
ATGGTGGTGAAACCCGCTTCGACGGTCAAATCATCGTTCAAATGGAAAACGACCCGG
TAGTAGCAAAAGCGGCAGCCAATTTAGCAGGTAAACATGCTGAAAGCAGTGTGGTGG
TGCAGCTCGATTCAGACGGCAACTATCGCGTGGTGTATGGCGATCCGTCAAAACTGG
ATGGAAAGCTACGTTGGCAGTTGGTGGGGCATGGTCGCGACCACTCAGAAACTAAC
AATACTCGCTTAAGTGGTTACAGTGCCGATGAGTTGGCCGTGAAATTGGCCAAGTTC
CAACAGTCGTTTAATCAAGCCGAAAACATCAACAACAAACCGGATCACATCAGTATTG
TTGGTTGTTCTTTGGTGAGTGACGACAAGCAAAAAGGCTTTGGTCATCAGTTTATTAA
CGCGATGGATGCGAATGGTCTTCGTGTCGATGTCTCTGTTCGTAGTTCTGAACTGGC
CGTAGACGAGGCGGGACGTAAGCATACCAAGGACGCGAATGGCGATTGGGTTCAAA
AGGCAGAAAACAACAAAGTTTCGCTAAGCTGGGACGCGCAAGGTCTCGAGCACCAC
CACCACCACCAC

4.4 EndoA-CPD

Protein sequence of CPD-tagged Endo-A:
MSTYNGPLSSHWFPEELAQWEPDSDPDAPFNRSHVPLEPGRVANRVNANADKDAHLV
SLSALNRHTSGVPSQGAPVFYENTFSYWHYTDLMVYWAGSAGEGIIVPPSADVIDASHR
NGVPILGNVFFPPTVYGGQLEWLEQMLEQEEDGSFPLADKLLEVADYYGFDGWFINQE
TEGADEGTAEAMQAFLVYLQEQKPEGMHIMWYDSMIDTGAIAWQNHLTDRNKMYLQN
GSTRVADSMFLNFWWRDQRQSNELAQALGRSPYDLYAGVDVEARGTSTPVQWEGLFP
EGEKAHTSLGLYRPDWAFQSSETMEAFYEKELQFWVGSTGNPAETDGQSNWPGMAH
WFPAKSTATSVPFVTHFNTGSGAQFSAEGKTVSEQEWNNRSLQDVLPTWRWIQHGGD
LEATFSWEEAFEGGSSLQWHGSLAEGEHAQIELYQTELPISEGTSLTWTFKSEHGNDLN
VGFRLDGEEDFRYVEGEQRESINGWTQWTLPLDAFAGQTITGLAFAAEGNETGLAEFYI
GQLAVGADSEKPAAPNVNVRQYDPDPSGIQLVWEKQSNVHHYRVYKETKHGKELIGTS
AGDRIYLEGLVEESKQNDVRLHIEALSETFVPSDARMIDIKSGSFVDALADGKILHNQNVN
SWGPITVTPTTDGGETRFDGQIIVQMENDPVVAKAAANLAGKHAESSVVVQLDSDGNY
RVVYGDPSKLDGKLRWQLVGHGRDHSETNNTRLSGYSADELAVKLAKFQQSFNQAENI
NNKPDHISIVGCSLVSDDKQKGFGHQFINAMDANGLRVDVSVRSSELAVDEAGRKHTKD
ANGDWVQKAENNKVSLSWDAQGLEHHHHHH

DNA sequence of CPD-tagged Endo-A:
ATGTCTACGTACAACGGCCCGCTGTCCTCCCATTGGTTTCCAGAGGAACTTGCCCAA
TGGGAACCAGACAGTGATCCAGACGCACCCTTTAACAGAAGCCATGTTCCGCTGGA
ACCAGGCCGCGTTGCGAATAGGGTAAATGCTAATGCAGACAAGGACGCACACCTTG
TTTCGTTGTCCGCGCTAAACAGGCATACATCAGGTGTTCCATCGCAAGGAGCGCCAG
TTTTCTATGAAAATACGTTCAGCTATTGGCATTATACAGATTTGATGGTTTATTGGGCTG
GTTCAGCTGGCGAAGGCATTATCGTTCCGCCAAGTGCCGATGTCATTGATGCATCGC
ACCGAAATGGGGTGCCGATTTTAGGAAATGTGTTCTTCCCGCCGACGGTTTATGGAG
GGCAGCTAGAGTGGCTAGAACAAATGTTAGAGCAAGAGGAGGACGGTTCATTCCCC

CTTGCTGACAAATTGCTAGAAGTCGCAGACTATTATGGGTTTGACGGCTGGTTTATTA
ACCAAGAAACAGAAGGGGCAGACGAAGGAACAGCCGAAGCCATGCAAGCTTTTCTC
GTTTATTTGCAGGAACAAAAGCCAGAAGGCATGCACATCATGTGGTATGACTCGATGA
TTGATACAGGGGCGATCGCCTGGCAAAACCATTTAACGGATCGAAATAAAATGTACTT
GCAAAATGGCTCGACCCGCGTCGCTGACAGCATGTTTTTGAACTTTTGGTGGCGTG
ACCAGCGCCAATCGAACGAATTGGCACAAGCACTTGGCAGGTCTCCGTATGACCTCT
ATGCCGGAGTGGATGTGGAAGCACGAGGGACAAGTACCCCTGTTCAGTGGGAAGG
CCTGTTTCCTGAAGGAGAAAAGGCGCATACATCACTCGGGTTATACCGTCCAGATTG
GGCATTTCAGTCAAGTGAAACAATGGAAGCGTTTTATGAAAAAGAACTACAATTTTGG
GTTGGCTCGACAGGAAATCCAGCCGAAACAGACGGCCAGTCAAATTGGCCTGGCAT
GGCGCACTGGTTTCCCGCGAAAAGCACCGCTACTTCGGTACCCTTTGTGACTCACTT
TAATACGGGCAGCGGCGCTCAGTTTTCGGCAGAAGGCAAAACTGTGTCGGAACAGG
AATGGAATAACCGCAGCCTTCAAGATGTGCTGCCGACATGGCGCTGGATTCAGCATG
GCGGCGATTTAGAGGCAACATTTTCTTGGGAAGAAGCGTTTGAAGGGGGAAGCTCG
TTACAATGGCATGGCTCATTAGCGGAAGGAGAACACGCCCAAATCGAGCTCTATCAA
ACAGAGTTGCCGATAAGCGAAGGCACTTCGCTAACGTGGACATTTAAAAGCGAGCAC
GGCAACGATTTAAATGTGGGCTTCCGTTTAGATGGGGAAGAGGACTTCCGTTATGTG
GAAGGAGAACAGCGTGAATCGATAAATGGTTGGACGCAGTGGACGTTGCCGCTGGA
TGCGTTTGCTGGTCAGACGATAACAGGGCTGGCATTTGCAGCGGAAGGGAATGAGA
CTGGGCTGGCAGAATTCTATATTGGACAACTGGCCGTAGGTGCTGATAGCGAAAAGC
CTGCCGCTCCAAACGTGAACGTACGCCAGTACGACCCAGACCCGAGTGGCATTCAG
CTCGTATGGGAAAAACAAAGCAACGTCCACCATTACCGCGTTTATAAAGAAACAAAGC
ACGGCAAAGAGCTAATTGGCACATCTGCTGGAGATCGAATTTACCTAGAAGGCCTAG
TCGAGGAAAGCAAACAAAACGACGTGCGTCTGCATATAGAAGCACTAAGTGAAACAT
TTGTGCCAAGTGATGCTCGCATGATCGACATAAAAAGCGGCTCGTTTGTCGACGCAT
TAGCGGATGGAAAAATACTCCATAATCAAAATGTTAATAGCTGGGGCCCGATTACGGT
TACACCAACGACAGATGGTGGTGAAACCCGCTTCGACGGTCAAATCATCGTTCAAAT
GGAAAACGACCCGGTAGTAGCAAAAGCGGCAGCCAATTTAGCAGGTAAACATGCTG
AAAGCAGTGTGGTGGTGCAGCTCGATTCAGACGGCAACTATCGCGTGGTGTATGGC
GATCCGTCAAAACTGGATGGAAAGCTACGTTGGCAGTTGGTGGGGCATGGTCGCGA
CCACTCAGAAACTAACAATACTCGCTTAAGTGGTTACAGTGCCGATGAGTTGGCCGT
GAAATTGGCCAAGTTCCAACAGTCGTTTAATCAAGCCGAAAACATCAACAACAAACC
GGATCACATCAGTATTGTTGGTTGTTCTTTGGTGAGTGACGACAAGCAAAAAGGCTTT
GGTCATCAGTTTATTAACGCGATGGATGCGAATGGTCTTCGTGTCGATGTCTCTGTTC
GTAGTTCTGAACTGGCCGTAGACGAGGCGGGACGTAAGCATACCAAGGACGCGAAT
GGCGATTGGGTTCAAAAGGCAGAAAACAACAAAGTTTCGCTAAGCTGGGACGCGCA
AGGTCTCGAGCACCACCACCACCACCAC

4.5 EndoS2-CPD

Protein sequence of CPD-tagged Endo-S2:
MEKTVQTGKTDQQVGAKLVQEIREGKRGPLYAGYFRTWHDRASTGIDGKQQHPENTM
AEVPKEVDILFVFHDHTASDSPFWSELKDSYVHKLHQQGTALVQTIGVNELNGRTGLSK
DYPDTPEGNKALAAAIVKAFVTDRGVDGLDIDIEHEFTNKRTPEEDARALNVFKEIAQLIG
KNGSDKSKLLIMDTTLSVENNPIFKGIAEDLDYLLRQYYGSQGGEAEVDTINSDWNQYQ

NYIDASQFMIGFSFFEESASKGNLWFDVNEYDPNNPEKGKDIEGTRAKKYAEWQPSTG
GLKAGIFSYAIDRDGVAHVPSTYKNRTSTNLQRHEVDNISHTDYTVSRKLKTLMTEDKRY
DVIDQKDIPDPALREQIIQQVGQYKGDLERYNKTLVLTGDKIQNLKGLEKLSKLQKLELRQ
LSNVKEITPELLPESMKKDAELVMVGMTGLEKLNLSGLNRQTLDGIDVNSITHLTSFDISH
NSLDLSEKSEDRKLLMTLMEQVSNHQKITVKNTAFENQKPKGYYPQTYDTKEGHYDVD
NAEHDILTDFVFGTVTKRNTFIGDEEAFAIYKEGAVDGRQYVSKDYTYEAFRKDYKGYKV
HLTASNLGETVTSKVTATTDETYLVDVSDGEKVVHHMKLNIGSGAIMMENLAKGAKVIGT
SGDFEQAKKIFDGEKSDRFFTWGQTNWIAFDLGEINLAKEWRLFNAETNTEIKTDSSLN
VAKGRLQILKDTTIDLEKMDIKNRKEYLSNDENWTDVAQMDDAKAVDALADGKILHNQNV
NSWGPITVTPTTDGGETRFDGQIIVQMENDPVVAKAAANLAGKHAESSVVVQLDSDGN
YRVVYGDPSKLDGKLRWQLVGHGRDHSETNNTRLSGYSADELAVKLAKFQQSFNQAE
NINNKPDHISIVGCSLVSDDKQKGFGHQFINAMDANGLRVDVSVRSSELAVDEAGRKHT
KDANGDWVQKAENNKVSLSWDAQGLEHHHHHH

DNA sequence of CPD-tagged Endo-S2:
ATGGAAAAAACCGTGCAGACCGGTAAAACCGATCAGCAGGTTGGCGCTAAACTGGT
GCAGGAAATTCGCGAAGGTAAACGTGGTCCGCTGTACGCGGGTTACTTCCGTACCT
GGCACGATCGCGCGTCGACCGGTATCGACGGTAAACAGCAGCACCCGGAAAACAC
CATGGCAGAAGTCCCGAAAGAAGTTGACATCCTGTTCGTTTTCCATGATCACACTGC
CAGCGATAGCCCGTTCTGGTCTGAACTGAAAGACAGCTACGTGCACAAACTGCACC
AGCAGGGTACCGCGCTGGTGCAGACCATTGGCGTTAACGAACTGAACGGCCGTACC
GGCCTGAGCAAAGATTACCCGGACACGCCGGAAGGCAACAAAGCACTGGCAGCTG
CGATCGTGAAAGCCTTCGTTACGGACCGCGGCGTTGATGGCCTGGATATTGATATCG
AACATGAATTCACTAACAAACGTACCCCGGAAGAAGATGCGCGTGCGCTGAACGTTT
TCAAAGAAATCGCACAGCTGATCGGCAAAAACGGCTCTGATAAATCTAAACTGCTGAT
CATGGATACCACCCTGTCCGTTGAAAATAACCCGATTTTTAAAGGTATCGCCGAGGAT
CTGGATTATCTTCTGCGTCAGTATTACGGCTCCCAGGGTGGCGAAGCCGAAGTTGAT
ACCATTAACAGCGATTGGAACCAGTACCAGAACTACATCGATGCATCTCAGTTCATGA
TCGGCTTCTCCTTCTTCGAAGAATCCGCGAGCAAAGGTAACCTGTGGTTTGATGTTA
ACGAGTATGACCCGAACAACCCGGAAAAAGGTAAAGACATCGAAGGTACCCGTGCC
AAAAAATACGCGGAATGGCAGCCGTCTACCGGCGGCCTGAAAGCGGGTATCTTCAG
CTACGCTATCGACCGCGACGGCGTCGCTCACGTGCCATCTACCTATAAAAACCGTAC
CTCCACCAACCTGCAGCGTCACGAAGTCGATAACATTAGCCACACTGATTACACCGT
GAGCCGTAAACTGAAAACCCTGATGACCGAAGATAAACGTTACGACGTTATTGACCA
GAAAGATATCCCGGATCCGGCTCTGCGTGAACAGATCATCCAGCAAGTGGGCCAGTA
CAAAGGTGATCTGGAACGTTACAACAAAACCCTGGTGCTGACCGGCGATAAAATTCA
GAATCTGAAAGGTCTGGAAAAACTGTCTAAACTGCAGAAACTGGAACTGCGTCAGCT
GTCCAACGTGAAGGAAATCACGCCGGAACTGCTGCCGGAATCCATGAAAAAAGACG
CGGAGCTGGTGATGGTGGGTATGACCGGTCTGGAAAAACTGAACCTGAGCGGTCTG
AACCGTCAGACGCTGGATGGCATCGATGTTAATAGCATCACCCACCTGACCTCGTTT
GACATTTCCCATAACTCTCTGGATCTGAGCGAAAAATCCGAGGACCGTAAACTGCTG
ATGACCCTGATGGAACAGGTTTCTAACCACCAGAAAATCACCGTTAAAAACACTGCG
TTCGAAAACCAGAAACCGAAAGGTTACTATCCGCAGACTTATGACACCAAAGAAGGC
CACTACGATGTTGACAACGCTGAACATGATATCCTGACCGACTTCGTTTTTGGCACC

GTGACTAAACGTAACACCTTCATTGGTGACGAAGAGGCGTTTGCGATCTATAAAGAA
GGCGCGGTTGATGGTCGTCAGTATGTAAGCAAAGACTACACCTATGAAGCGTTCCGC
AAAGACTATAAAGGCTATAAAGTGCACCTGACTGCCTCCAACCTGGGTGAAACCGTA
ACCTCTAAAGTGACCGCGACGACTGATGAAACCTACCTGGTTGATGTTAGTGATGGC
GAAAAAGTGGTTCACCACATGAAACTGAACATTGGCTCCGGCGCGATTATGATGGAA
AACCTGGCTAAAGGCGCTAAAGTTATTGGTACTTCTGGCGACTTCGAACAGGCTAAG
AAAATCTTCGACGGCGAAAAAAGCGATCGCTTCTTCACCTGGGGCCAGACCAACTG
GATCGCGTTTGATCTGGGTGAAATTAACCTGGCGAAAGAGTGGCGTTTGTTCAACGC
GGAAACCAACACTGAAATCAAAACCGACAGCAGCCTGAACGTTGCGAAAGGTCGTC
TGCAGATCCTGAAAGACACCACCATCGACCTGGAAAAAATGGACATCAAAAATCGTA
AAGAATATCTGAGCAACGATGAAAACTGGACCGACGTGGCACAGATGGATGATGCTA
AAGCTGTCGACGCATTAGCGGATGGAAAAATACTCCATAATCAAAATGTTAATAGCTG
GGGCCCGATTACGGTTACACCAACGACAGATGGTGGTGAAACCCGCTTCGACGGTC
AAATCATCGTTCAAATGGAAAACGACCCGGTAGTAGCAAAAGCGGCAGCCAATTTAG
CAGGTAAACATGCTGAAAGCAGTGTGGTGGTGCAGCTCGATTCAGACGGCAACTATC
GCGTGGTGTATGGCGATCCGTCAAAACTGGATGGAAAGCTACGTTGGCAGTTGGTG
GGGCATGGTCGCGACCACTCAGAAACTAACAATACTCGCTTAAGTGGTTACAGTGCC
GATGAGTTGGCCGTGAAATTGGCCAAGTTCCAACAGTCGTTTAATCAAGCCGAAAAC
ATCAACAACAAACCGGATCACATCAGTATTGTTGGTTGTTCTTTGGTGAGTGACGACA
AGCAAAAAGGCTTTGGTCATCAGTTTATTAACGCGATGGATGCGAATGGTCTTCGTGT
CGATGTCTCTGTTCGTAGTTCTGAACTGGCCGTAGACGAGGCGGGACGTAAGCATAC
CAAGGACGCGAATGGCGATTGGGTTCAAAAGGCAGAAAACAACAAAGTTTCGCTAA
GCTGGGACGCGCAAGGTCTCGAGCACCACCACCACCACCAC

4.6 Her2 Nb-CPD (flag tag)

Protein sequence of CPD-tagged Her2 nanobody (flag tag):
MDYKDDDDKQVQLQESGGGSVQAGGSLKLTCAASGYIFNSCGMGWYRQSPGREREL
VSRISGDGDTWHKESVKGRFTISQDNVKKTLYLQMNSLKPEDTAVYFCAVCYNLETYWG
QGTQVTVSSGGVDALADGKILHNQNVNSWGPITVTPTTDGGETRFDGQIIVQMENDPV
VAKAAANLAGKHAESSVVVQLDSDGNYRVVYGDPSKLDGKLRWQLVGHGRDHSETNN
TRLSGYSADELAVKLAKFQQSFNQAENINNKPDHISIVGCSLVSDDKQKGFGHQFINAMD
ANGLRVDVSVRSSELAVDEAGRKHTKDANGDWVQKAENNKVSLSWDAQGLEHHHHH
H

DNA sequence of CPD-tagged Her2 nanobody (flag tag):
ATGGATTACAAGGACGACGATGACAAGCAAGTTCAGCTGCAGGAAAGCGGTGGTGG
TAGCGTTCAGGCCGGTGGTAGCCTGAAACTGACCTGCGCCGCCAGCGGTTATATTTT
TAATAGCTGCGGTATGGGTTGGTATCGTCAGAGTCCGGGTCGTGAACGTGAACTGGT
TAGCCGTATTAGCGGTGATGGTGATACCTGGCATAAAGAAAGCGTGAAAGGTCGTTT
TACCATTAGCCAGGATAATGTGAAGAAAACCCTGTATCTGCAGATGAATAGCCTGAAA
CCGGAAGATACCGCCGTGTATTTTTGCGCCGTTTGCTATAATCTGGAAACCTATTGGG
GTCAGGGTACCCAGGTGACCGTGAGCAGCGGTGGTGTCGACGCATTAGCGGATGG
AAAAATACTCCATAATCAAAATGTTAATAGCTGGGGCCCGATTACGGTTACACCAACG
ACAGATGGTGGTGAAACCCGCTTCGACGGTCAAATCATCGTTCAAATGGAAAACGAC

CCGGTAGTAGCAAAAGCGGCAGCCAATTTAGCAGGTAAACATGCTGAAAGCAGTGTG
GTGGTGCAGCTCGATTCAGACGGCAACTATCGCGTGGTGTATGGCGATCCGTCAAA
ACTGGATGGAAAGCTACGTTGGCAGTTGGTGGGGCATGGTCGCGACCACTCAGAAA
CTAACAATACTCGCTTAAGTGGTTACAGTGCCGATGAGTTGGCCGTGAAATTGGCCA
AGTTCCAACAGTCGTTTAATCAAGCCGAAAACATCAACAACAAACCGGATCACATCAG
TATTGTTGGTTGTTCTTTGGTGAGTGACGACAAGCAAAAAGGCTTTGGTCATCAGTTT
ATTAACGCGATGGATGCGAATGGTCTTCGTGTCGATGTCTCTGTTCGTAGTTCTGAAC
TGGCCGTAGACGAGGCGGGACGTAAGCATACCAAGGACGCGAATGGCGATTGGGTT
CAAAAGGCAGAAAACAACAAAGTTTCGCTAAGCTGGGACGCGCAAGGTCTCGAGCA
CCACCACCACCACCAC

5. NMR spectra

^{1}H NMR of compound **S1**

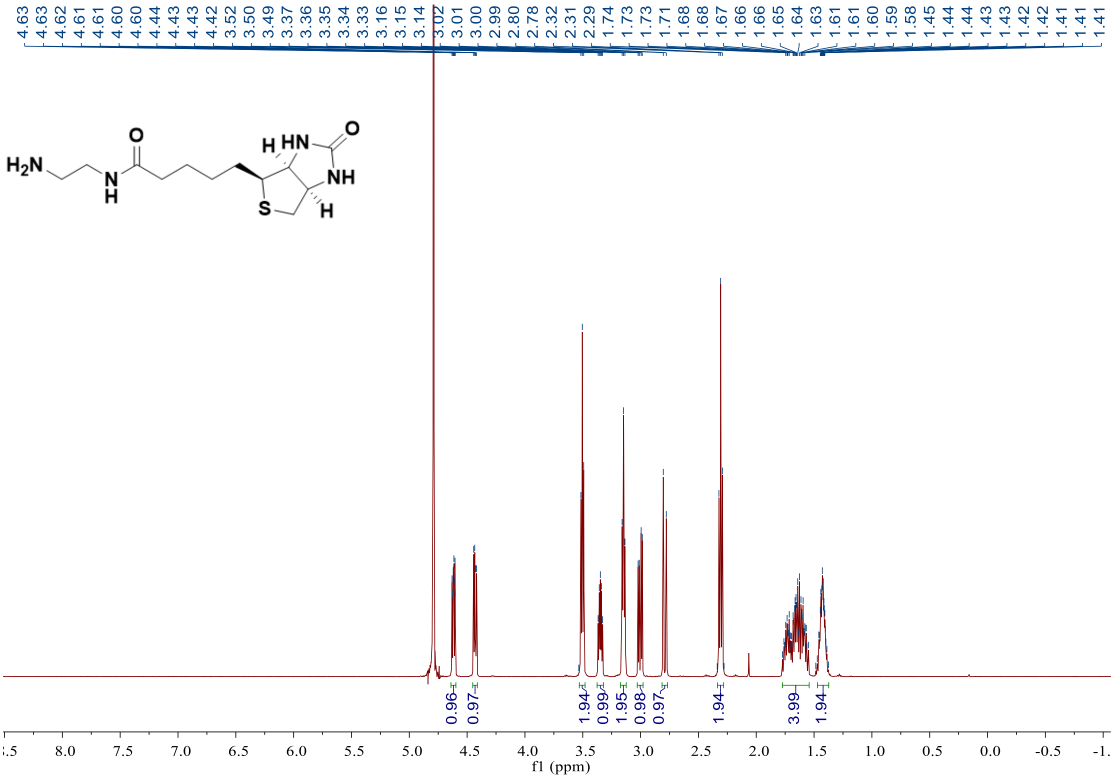

^{13}C NMR of compound **S1**

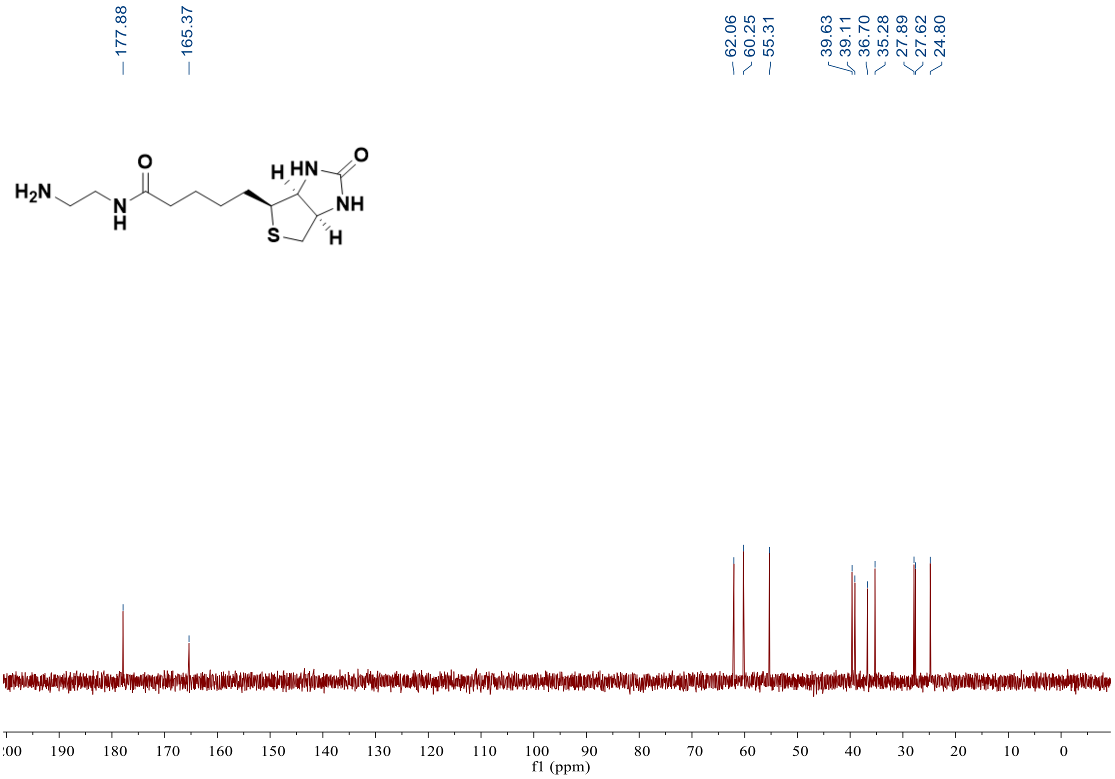

^{1}H NMR of compound **S2**

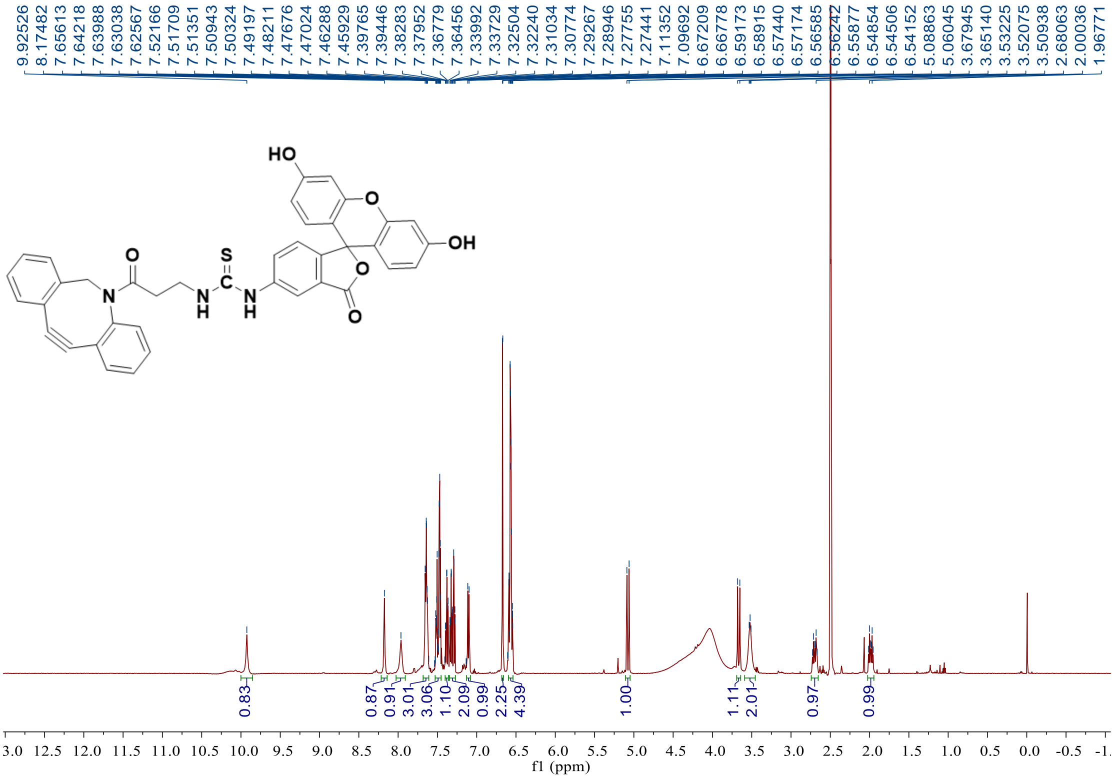

^{13}C NMR of compound **S2**

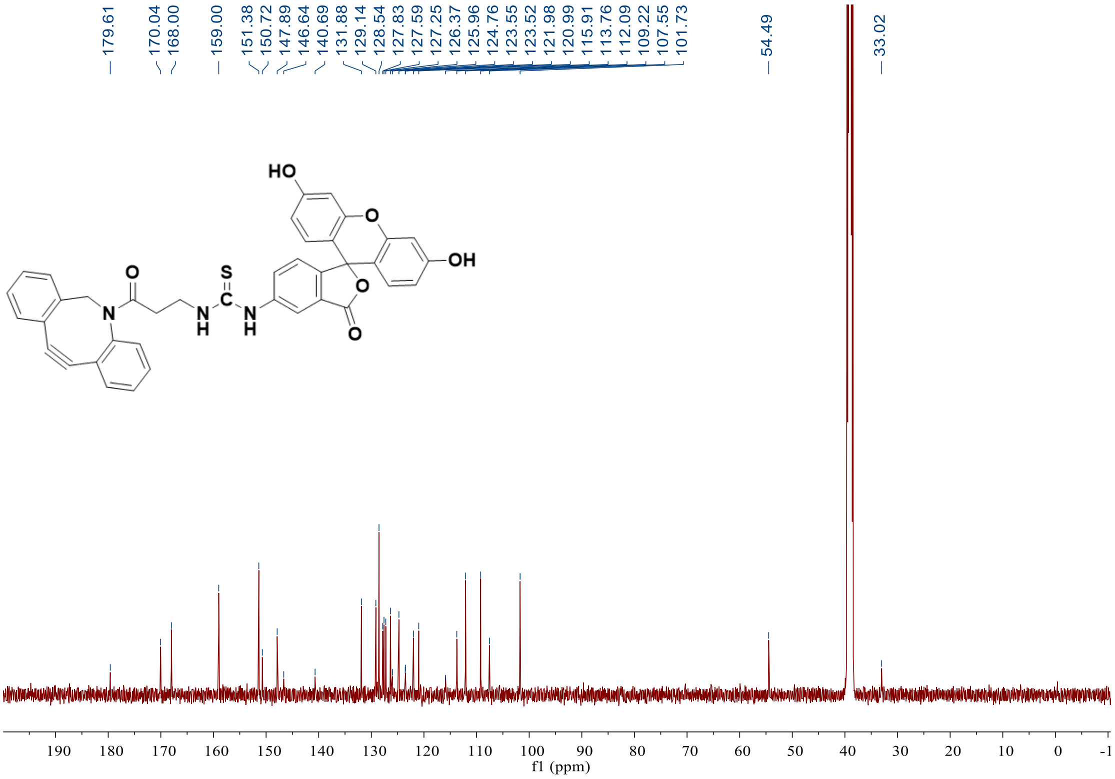

^{1}H NMR of compound **S11**

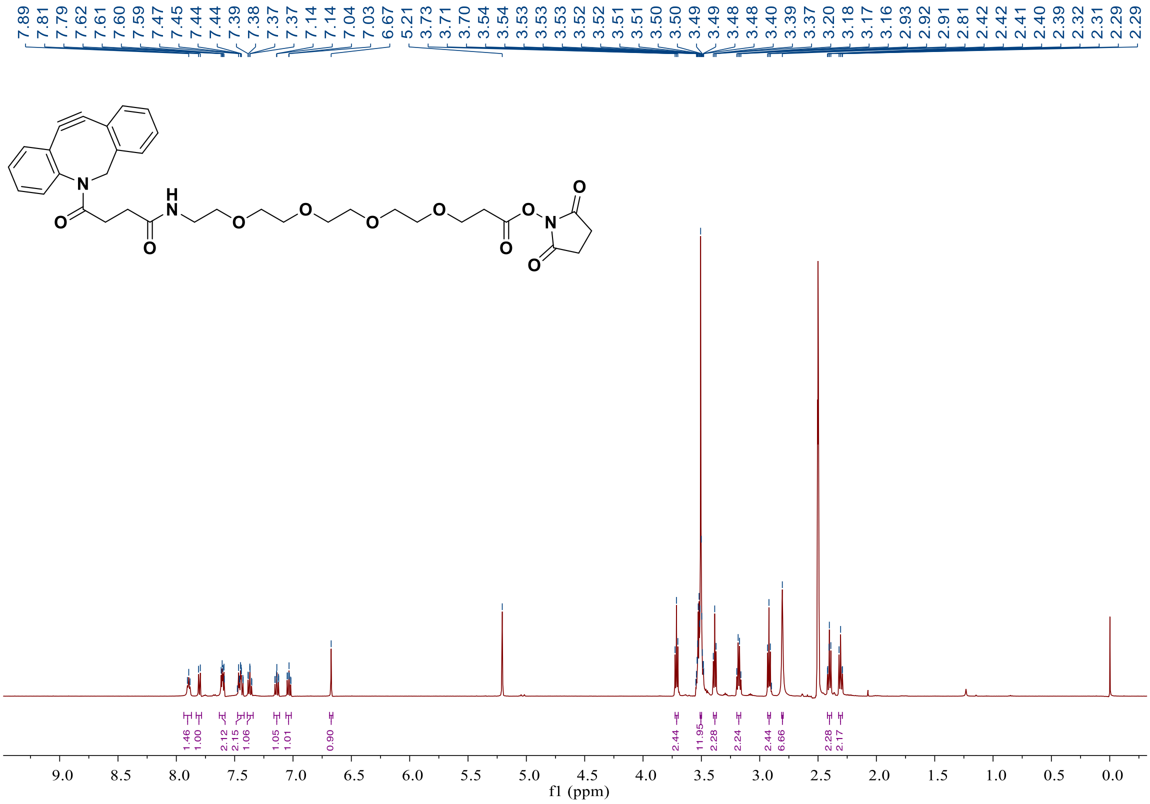

^{13}C NMR of compound **S11**

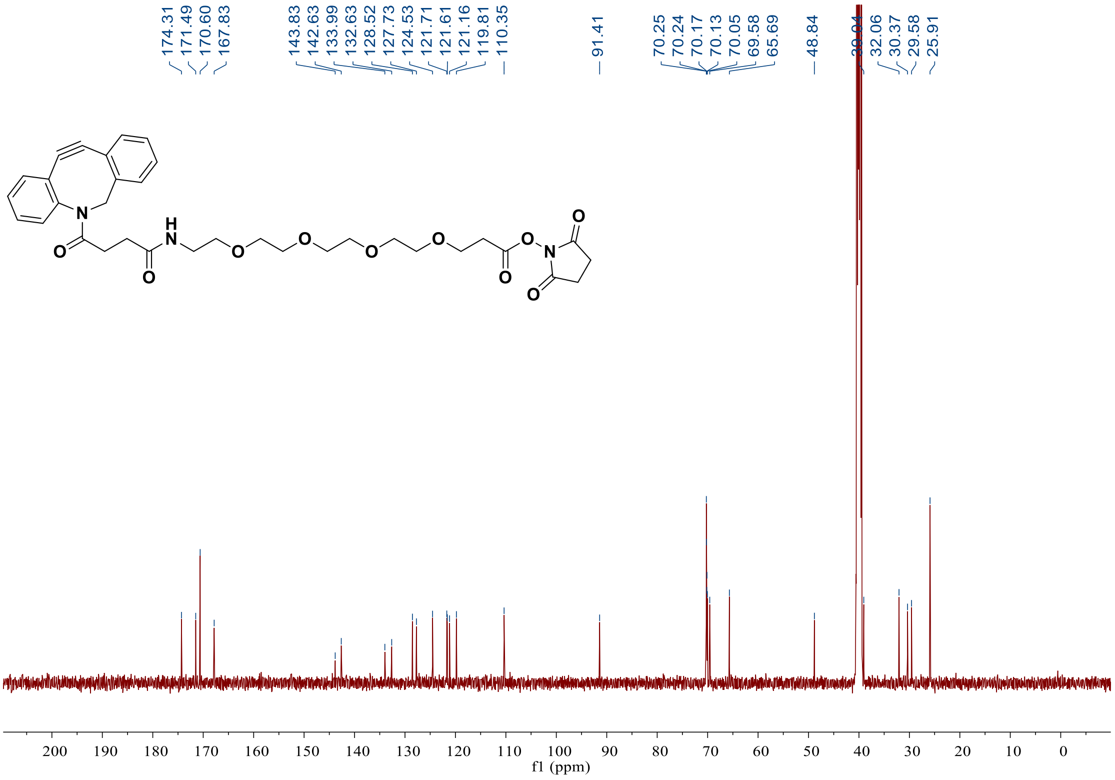

Supplement: Supplementary file 1 — Supplementary Information [file 41467_2023_42977_MOESM1_ESM.pdf]
