# Supplementary material for: C-terminal modification and functionalization of proteins via a self-cleavage tag triggered by a small molecule
Source: Nat Commun. 2023 Nov 7;14:7169. doi: 10.1038/s41467-023-42977-x (PMC10630284; doi:10.1038/s41467-023-42977-x)
Supplement: Supplementary file 3 — Reporting Summary [file 41467_2023_42977_MOESM3_ESM.pdf]

nature portfolio

Corresponding author(s): Feng Tang

Last updated by author(s): Sep 30, 2023

Reporting Summary

Nature Portfolio wishes to improve the reproducibility of the work that we publish. This form provides structure for consistency and transparency in reporting. For further information on Nature Portfolio policies, see our Editorial Policies and the Editorial Policy Checklist.

Statistics

For all statistical analyses, confirm that the following items are present in the figure legend, table legend, main text, or Methods section.

n/a	Confirmed	
☐	☒	The exact sample size (*n*) for each experimental group/condition, given as a discrete number and unit of measurement
☐	☒	A statement on whether measurements were taken from distinct samples or whether the same sample was measured repeatedly
☒	☐	The statistical test(s) used AND whether they are one- or two-sided<br>*Only common tests should be described solely by name; describe more complex techniques in the Methods section.*
☒	☐	A description of all covariates tested
☐	☒	A description of any assumptions or corrections, such as tests of normality and adjustment for multiple comparisons
☐	☒	A full description of the statistical parameters including central tendency (e.g. means) or other basic estimates (e.g. regression coefficient) AND variation (e.g. standard deviation) or associated estimates of uncertainty (e.g. confidence intervals)
☒	☐	For null hypothesis testing, the test statistic (e.g. *F*, *t*, *r*) with confidence intervals, effect sizes, degrees of freedom and *P* value noted<br>*Give P values as exact values whenever suitable.*
☒	☐	For Bayesian analysis, information on the choice of priors and Markov chain Monte Carlo settings
☒	☐	For hierarchical and complex designs, identification of the appropriate level for tests and full reporting of outcomes
☒	☐	Estimates of effect sizes (e.g. Cohen's *d*, Pearson's *r*), indicating how they were calculated

Our web collection on statistics for biologists contains articles on many of the points above.

Software and code

Policy information about availability of computer code

Data collection	Waters Xevo G2-XS Q-TOF; Bruker Ascend-400/500; Thermo ultimate 3000; Beijing ChuangXinTongHeng LC3000; Leica TCS-SP8 STED
Data analysis	Waters UNIFI; MestReNova 9.0.1; Chromeleon 7; LabChrom; Leica Application Suite X; Image Lab 5.2.1; Prism 8; Origin 2022; SnapGene 3.2.1

For manuscripts utilizing custom algorithms or software that are central to the research but not yet described in published literature, software must be made available to editors and reviewers. We strongly encourage code deposition in a community repository (e.g. GitHub). See the Nature Portfolio guidelines for submitting code & software for further information.

Data

Policy information about availability of data

All manuscripts must include a data availability statement. This statement should provide the following information, where applicable:

- Accession codes, unique identifiers, or web links for publicly available datasets
- A description of any restrictions on data availability
- For clinical datasets or third party data, please ensure that the statement adheres to our policy

All data sets were contained in the manuscript or the Supplementary material. A Source Data file has been placed alongside the manuscript. No clinical datasets or third party data were contained in the manuscript.

Research involving human participants, their data, or biological material

Policy information about studies with human participants or human data. See also policy information about sex, gender (identity/presentation), and sexual orientation and race, ethnicity and racism.

Reporting on sex and gender	not applicable
Reporting on race, ethnicity, or other socially relevant groupings	not applicable
Population characteristics	not applicable
Recruitment	not applicable
Ethics oversight	not applicable

Note that full information on the approval of the study protocol must also be provided in the manuscript.

Field-specific reporting

Please select the one below that is the best fit for your research. If you are not sure, read the appropriate sections before making your selection.

[x] Life sciences [ ] Behavioural & social sciences [ ] Ecological, evolutionary & environmental sciences

For a reference copy of the document with all sections, see nature.com/documents/nr-reporting-summary-flat.pdf

Life sciences study design

All studies must disclose on these points even when the disclosure is negative.

Sample size	No sample size calculations were performed. Sample size was chosen based on established practice in the field and was sufficient as the results were reproducible.
Data exclusions	No data was excluded from the analysis.
Replication	All measurements were done at least in triplicate and all attempts at replication were successful and presented.
Randomization	There were no experiments for randomization.
Blinding	Blinding was not relevant to this study as no investigator-dependent selection of data was performed.

Reporting for specific materials, systems and methods

We require information from authors about some types of materials, experimental systems and methods used in many studies. Here, indicate whether each material, system or method listed is relevant to your study. If you are not sure if a list item applies to your research, read the appropriate section before selecting a response.

Materials & experimental systems

n/a	Involved in the study	
[ ]	[x]	Antibodies
[ ]	[x]	Eukaryotic cell lines
[x]	[ ]	Palaeontology and archaeology
[x]	[ ]	Animals and other organisms
[x]	[ ]	Clinical data
[x]	[ ]	Dual use research of concern
[x]	[ ]	Plants

Methods

n/a	Involved in the study	
[x]	[ ]	ChIP-seq
[x]	[ ]	Flow cytometry
[x]	[ ]	MRI-based neuroimaging

Antibodies

Antibodies used	CPD-tagged Her2 nanobodies were expressed in house (see Supplementary information). Trastuzumab (cat. BDS15-20210301) was ordered and produced by TOT Biopharm. These two antibodies were used as reactants. We didn't use trastuzumab in any assay experiments. We synthesized the Nb-FITC conjugate in house and diluted it into 10 ug/mL in wash buffer (PBS containing 5% FBS), 1: 100 dilution from stock (1 mg/mL).
Validation	CPD-tagged Her2 nanobodies: validated for purity and molecular weight by ESI-MS, RP-HPLC, SDS-PAGE and HIC analysis, validated in

house for specificity by ELISA. Trastuzumab: used only for conjugation, validated for molecular weight by ESI-MS, HIC, and SEC analysis, not validated for antigen specificity.

Eukaryotic cell lines

Policy information about cell lines and Sex and Gender in Research

Cell line source(s)	SK-Br-3 and MDA-MB-231 was obtained from Cell Bank of Chinese Academy of Sciences (Shanghai, China).
Authentication	ELISA for Her2 binding (SK-Br-3, MDA-MB-231).
Mycoplasma contamination	All cell lines were periodically tested for mycoplasma contamination and tested negative.
Commonly misidentified lines (See ICLAC register)	None
